# Sphingolipids as New Biomarkers for Assessment of Delayed-Type Hypersensitivity and Response to Triptolide

**DOI:** 10.1371/journal.pone.0052454

**Published:** 2012-12-26

**Authors:** Feng Qu, Cai-Sheng Wu, Jin-Feng Hou, Ying Jin, Jin-Lan Zhang

**Affiliations:** State Key Laboratory of Bioactive Substance and Function of Natural Medicines, Institute of Materia Medica, Peking Union Medical College and Chinese Academy of Medical Sciences, Beijing, China; MUSC SC College of Pharmacy, United States of America

## Abstract

**Background:**

Hypersensitivity diseases are associated with many severe human illnesses, including leprosy and tuberculosis. Emerging evidence suggests that the pathogenesis and pathological mechanisms of treating these diseases may be attributable to sphingolipid metabolism.

**Methods:**

High performance liquid chromatography-tandem mass spectrometry was employed to target and measure 43 core sphingolipids in the plasma, kidneys, livers and spleens of BALB/c mice from four experimental groups: control, delayed-type hypersensitivity (DTH) model, DTH+triptolide, and control+triptolide. Orthogonal partial least squares discriminant analysis (OPLS-DA) was used to identify potential biomarkers associated with variance between groups. Relationships between the identified biomarkers and disease markers were evaluated by Spearman correlation.

**Results:**

As a treatment to hypersensitivity disease, triptolide significantly inhibit the ear swelling and recover the reduction of splenic index caused by DTH. The sphingolipidomic result revealed marked alterations in sphingolipid levels between groups that were associated with the effects of the disease and triptolide treatment. Based on this data, 23 potential biomarkers were identified by OPLS-DA, and seven of these biomarkers correlated markedly with the disease markers (p<0.05) by Spearman correlation.

**Conclusions:**

These data indicate that differences in sphingolipid levels in plasma and tissues are related to DTH and treatment with triptolide. Restoration of proper sphingolipid levels may attribute to the therapeutic effect of triptolide treatment. Furthermore, these findings demonstrate that targeted sphingolipidomic analysis followed by multivariate analysis presents a novel strategy for the identification of biomarkers in biological samples.

## Introduction

**Figure 1:**
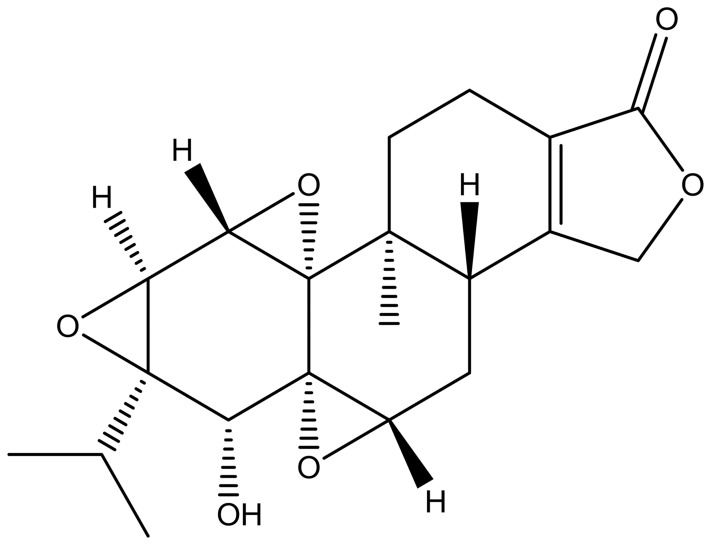
Structure of triptolide.

Type IV hypersensitivity is often called delayed-type hypersensitivity (DTH) as the reaction takes 2 to 3 days to develop. Unlike other types of hypersensitivity, which are antibody-mediated, DTH is a cell-mediated response. The pathogenesis of clinical diseases such as temporal arteritis, Hashimoto's thyroiditis, celiac disease, graft-versus-host disease, chronic transplant rejection and symptoms of leprosy and tuberculosis is associated with DTH [1]–[3]. DTH is an overreaction of the autoimmune system and triptolide can be used to treat DTH by suppressing this immune reaction [4]. Extracts of the Chinese traditional herb *Tripterygium wilfordii* Hook. f. (TWHF) are widely used in the treatment of autoimmune and inflammatory diseases, including systemic lupus erythematosus, rheumatoid arthritis, and dermatomyositis, and decrease rejection after tissue and organ transplantation [5]–[7]. Triptolide *(* ***Figure 1*** *),* the active compound in TWHF, exerts strong immunosuppressive activity *in vitro* and *in vivo* [8]–[14]. As a clinically utilized drug in China, treatment with TWHF and triptolide analogs must be administered with caution due to the nephrotoxic and hepatotoxic effects observed even at standard treatment doses [15], [16]. The toxicology of triptolide is an active area of biomedical research.

Sphingolipids are highly bioactive compounds that serve not only as core components of biological structures, such as membranes and lipoproteins, but also as regulators of cell proliferation, differentiation, cellular interactions and migration, intracellular (and extracellular) signaling, membrane trafficking, autophagy, and cell death [17]. Because of the highly integrated metabolism of many bioactive sphingolipids, abnormal changes in the content or activity of functional sphingolipids, even those within minor subclasses, can result in a ripple effect, causing inflammation, allergy, and tumor development [18]–[20]. Many researchers report that autoimmune and inflammatory diseases are tightly connected with sphingolipid activity [21], [22]. Systematic study of these connections may provide new insight into the mechanisms by which sphingolipids contribute to disease progression or to their utility as biomarkers of these diseases. Such studies will also be beneficial for the diagnosis and treatment of autoimmune and inflammatory diseases. The connections between sphingolipids and DTH or between sphingolipids and triptolide with regard to the etiology of DTH and the mechanism of action of triptolide remain to be elucidated.

As a branch of lipidomics, sphingolipidomics focuses on the large-scale study of sphingolipids and the pathways and networks they are involved [17], [23]–[25]. Mass spectrometry, especially high performance liquid chromatography-tandem mass spectrometry (HPLC-MS/MS), is a powerful tool for the analysis of sphingolipids due to its high specificity and sensitivity [26], [27], and has contributed significantly to understanding how sphingolipid biosynthesis and turnover regulates cell behavior under normal and abnormal conditions [18], [19]. The primary purpose of this study was to perform a targeted sphingolipidomic assay of the plasma and tissues from BALB/c mice in control, DTH, DTH+triptolide and control+triptolide groups in order to correlate the observed changes in sphingolipid levels with disease progression and treatment effectiveness. Based on a previously established LC-MS/MS method for profiling sphingolipids, we developed a fully-validated sphingolipidomic platform for profiling the sphingolipid composition in plasma, spleen, liver and kidney. Integrated with the results of our targeted sphingolipidomic study, we employed supervised orthogonal partial least squares discriminant analysis (OPLS-DA) to identify potential biomarkers and provide an improved mechanistic understanding of DTH and the effects of triptolide treatment in plasma and tissues, including the nephrotoxic effects of triptolide. Seven of the identified potential sphingolipid biomarkers correlated markedly with the severity of DTH, indicating that the development of DTH and the effect of triptolide treatment are connected with alteration of those sphingolipids.

## Materials and Methods

### 2.1 Materials

Ultra Resi-analyzed grade methanol and HPLC grade methyl-tert-butyl ether (MTBE) were purchased from Mallinckrodt Baker Inc. (Phillipsburg, NJ, USA). Formic acid of analytical grade was obtained from TEDIA Company, Inc. (Fairfield, OH, USA). Ammonium formate (purity >99.99%) was purchased from Sigma-Aldrich (St. Louis, MO, USA). Ultra-pure water was prepared using a Milli-Q purification system (Millipore, Bedford, MA, USA). Protease- and fatty-acid-free lyophilized bovine serum albumin (BSA) was purchased from SERVA (Heidelberg, Germany). Sodium chloride was from Merck (Darmstadt, Germany). Sodium hydroxide was obtained from Sinopharm Chemical Reagent Co. Ltd (Beijing, China). All sphingolipid standards were from Avanti Polar Lipids (Alabaster, AL, USA). 2,4-dinitrofluorobenzene (DNFB) was generously provided by Professor Hou (Chinese Academy of Medical Sciences & Peking Union Medical College, Beijing 100050, China). Triptolide (C_20_H_24_O_6_, molecular weight 360, 98%; ***Figure 1***) was purchased from Sigma-Aldrich, Inc (St. Louis, MO, USA), and prepared as described previously [28]. Brieﬂy, 1 mg triptolide was reconstituted in 2 mL dimethyl sulfoxide and freshly diluted with normal saline to a final concentration of 7 µg/mL before use.

### 2.2 Animals

A total of 32 male BALB/c mice (6–7 weeks old, body weight = 22.3±1.3 g) were purchased from the Institute of Laboratory Animal Science, Chinese Academy of Medical Sciences. The animals were housed under specific pathogen-free conditions (12 h light/12 h dark photoperiod, 23±2°C, 55±5% relative humidity). All mice were allowed to acclimate for 2 weeks before experiments were initiated. Research was conducted in accordance with all institutional guidelines and ethics and approved by the Laboratories Institutional Animal Care and Use Committee of the Chinese Academy of Medical Sciences and Peking Union Medical College.

### 2.3 DNFB-induced DTH Response

The DTH assay was carried out as described previously [29], [30]. Mice were divided randomly into four groups of eight mice: group A as control, group B as DTH model mice, group C as DTH model mice treated with triptolide (70 µg/kg, i.p.), and group D as control mice treated with triptolide (70 µg/kg, i.p.). Briefly, the mice were initially sensitized on each hind foot on days 0 and 1 with 0.5% DNFB dissolved in acetone-olive oil at a 4∶1 ratio (groups B & C) or vehicle alone (groups A & D). On day 9, the mice were challenged with 0.4% DNFB (groups B & C) or vehicle (groups A & D) on both sides of their left ear. Triptolide (70 µg/kg, i.p.; groups C & D) or normal saline (0.01 mL/g, i.p.; groups A & B) was administered to mice 1 h before and 12 h after the challenge. At 30 h after the last challenge, the mice were dissected after euthanization and blood samples were collected using heparin as an anticoagulant to obtain plasma by centrifugation (750×g, 15 min). The livers, kidneys and spleens were immediately frozen in liquid nitrogen for 30 seconds and kept at −80°C until further analysis. Ear swelling was expressed as the difference between the weight of left and right ear patches obtained by 8 mm punches taken 30 h after challenge. The punches were obtained in a blinded manner. The splenic index was expressed as the ratio of spleen weight to mouse weight.

Tissue samples were homogenized in a buffer solution containing 0.025 M KCl, 0.05 M Tris, 0.25 M sucrose and 0.005 M EDTA. The pH was adjusted to 7.4 by drop-wise addition of 2 M HCl. The ratio of tissue to buffer solution was 1∶10. The homogenate was transferred to a 10 mL glass tube, and a 0.1 mL aliquot of the homogenate was used for lipid extraction.

### 2.4 HPLC-MS/MS Analysis

A three-phase solvent system was employed for sample preparation, including MTBE, methanol and water. For detailed procedures, please refer to Information S1, ***Item 1.1.*** For detailed procedures regarding standard curves and method validation, please refer to Information S1, ***Items 1.2, 1.5.***

HPLC-MS/MS analysis was performed using an Agilent 6410B Triple Quad mass spectrometer (Agilent Technologies, Inc. Santa Clara, CA) comprised of a triple quadrupole MS analyzer with an electrospray ionization (ESI) interface usable in either positive ionization or negative ionization mode and an Agilent 1200 RRLC system. Chromatographic separation was performed using a Spectra C8SR Column (150 × 3.0 mm; 3 µm particle size; Peeke Scientific, Redwood City, CA). Mobile Phase A consisted of 2 mM ammonium formate in water containing 0.2% formic acid. Mobile Phase B consisted of 1 mM ammonium formate in methanol containing 0.2% formic acid [31]. The parameters for electrospray ionization tandem mass spectrometry (ESI-MS/MS) were as follows: polarity = positive, gas temperature = 350°C, gas flow = 6 L/min, nebulizer = 15 psi, capillary = 4000V. Multiple reaction monitoring (MRM) was conducted using the characteristic precursor-to-product ion transition, optimized fragmentor voltages and collision energy as shown in ***Table 1*** **.** For detailed gradient elution programs, please refer to Information S1, ***Item 1.3.*** Representative MS/MS fragmentation patterns of sphingolipids and MRM chromatogram were shown in ***Figure S1*** and ***Figure S2***, respectively.

**Table 1:** ESI-MS MS/MS MRM and HPLC conditions for each sphingolipid.

Seg	Compound	RT (min)	Pre-ion (m/z)	Pro-ion (m/z)	Fragmentor (V)	CE (eV)	ISTD
**LC-MS/MS run 1: For sphingosine, sphingosine-1-phosphate, ceramides, ceramides-1-phosphate**							
1	Sph (d17∶1)	2.5	286.0	267.8	100	7	ISTD
	Sph (d18∶1)	2.7	300.3	282.9	90	3	Sph (d17∶1)
	Sph (d17∶1)-1-P	4.3	365.9	250.0	100	11	ISTD
	Sph (d18∶1)-1-P	5.1	380.3	264.1	110	12	Sph (d17∶1)-1-P
	Cer(d18∶1/2∶0)	6.9	342.1	324.1	100	2	ISTD
	Cer(d18∶1/2∶0)-1-P	8.7	422.0	264.1	100	29	Cer(d18∶1/8∶0)-1-P
	Cer(d18∶1/4∶0)	9.0	370.2	352.1	100	2	Cer(d18∶1/2∶0)
2	Cer(d18∶1/6∶0)	12.6	398.2	380.1	100	2	Cer(d18∶1/10∶0)
	Cer(d18∶1/8∶0)	15.7	426.3	408.2	140	3	Cer(d18∶1/10∶0)
	Cer(d18∶1/8∶0)-1-P	17.2	506.0	264.1	120	26	ISTD
	Cer(d18∶1/10∶0)	17.5	454.2	436.2	140	5	ISTD
	Cer(d18∶1/12∶0)	19.4	482.4	464.2	140	7	Cer(d18∶1/10∶0)
	Cer(d18∶1/14∶0)	21.5	510.4	492.1	120	6	Cer(d18∶1/10∶0)
	Cer(d18∶1/12∶0)-1-P	21.5	562.1	264.1	120	26	Cer(d18∶1/10∶0)
3	Cer(d18∶1/16∶0)	23.8	538.4	520.2	140	6	Cer(d17∶1/18∶0)
	Cer(d18∶1/18∶1)	24.6	564.4	546.2	130	6	Cer(d17∶1/18∶0)
	Cer(d17∶1/18∶0)	25.1	552.4	250.1	130	30	ISTD
	Cer(d18∶1/18∶0)	26.2	566.4	548.3	150	12	Cer(d17∶1/18∶0)
	Cer(d18∶1/16∶0)-1-P	26.4	618.1	263.9	110	31	Cer(d18∶1/8∶0)-1-P
	Cer(d18∶1/18∶1)-1-P	27.3	644.2	264.1	150	34	Cer(d18∶1/8∶0)-1-P
4	Cer(d18∶1/20∶0)	28.6	594.4	576.2	140	8	Cer(d17∶1/24∶1)
	Cer(d17∶1/24∶1)	30.3	634.5	616.6	140	10	ISTD
	Cer(d18∶1/22∶0)	31.3	622.5	604.2	140	8	Cer(d17∶1/24∶1)
	Cer(d18∶1/24∶1)	31.4	648.7	630.2	140	12	Cer(d17∶1/24∶1)
	Cer(d18∶1/24∶0)	33.8	650.7	632.3	140	8	Cer(d17∶1/24∶1)
	Cer(d18∶1/26∶1)*	34.1	676.8	658.3	140	12	Cer(d17∶1/24∶1)
	Cer(d18∶1/26∶0)*	36.6	678.8	660.3	140	8	Cer(d17∶1/24∶1)
**LC-MS/MS run 2: For dihydrosphingosine, dihydorsphingosine-1-phosphate, dihydroceramides and hexcosyl-sphingolipids**							
1	HexSph(d18∶1)	3.2	462.1	282.2	130	21	dhSph(d17∶0)
	dhSph(d17∶0)	3.5	287.9	110.0	270	11	ISTD
	dhSph(d18∶0)	4.2	302.1	284.2	120	5	dhSph(d17∶0)
	dhSph-1-P(d17∶0)	6.4	367.8	110.0	270	9	ISTD
	dhSph-1-P(d18∶0)	8.2	382.1	284.2	80	10	dhSph-1-P(d17∶0)
	dhCer(d18∶0/2∶0)	12.0	344.1	326.2	110	10	dhCer(d18∶0/8∶0)
	HexCer(d18∶1/8∶0)	15.9	588.2	264.2	100	36	dhCer(d18∶0/8∶0)
	dhCer(d18∶0/6∶0)	16.0	400.1	382.2	130	10	dhCer(d18∶0/8∶0)
	dhCer(d18∶0/8∶0)	17.5	428.2	410.1	150	15	ISTD
2	HexCer(d18∶1/12∶0)	18.7	644.2	264.1	150	36	Cer(d17∶1/18∶0)
	HexCer(d18∶1/16∶0)	21.7	700.2	264.1	140	36	Cer(d17∶1/18∶0)
	HexCer(d18∶1/18∶1)	22.2	726.1	264.2	160	36	Cer(d17∶1/18∶0)
	HexCer(d18∶1/18∶0)	23.2	728.2	264.1	150	39	Cer(d17∶1/18∶0)
	dhCer(d18∶0/16∶0)	23.4	540.2	522.4	160	18	Cer(d17∶1/18∶0)
	Cer(d17∶1/18∶0)	23.7	552.4	250.1	130	30	ISTD
	dhCer(d18∶0/18∶1)	24.0	566.2	548.4	140	18	Cer(d17∶1/18∶0)
	dhCer(d18∶0/18∶0)	25.0	568.2	550.3	140	18	Cer(d17∶1/18∶0)
3	HexCer(d18∶1/24∶1)	26.6	810.2	264.1	100	36	Cer(d17∶1/24∶1)
	Cer(d17∶1/24∶1)	27.4	634.5	616.6	140	10	ISTD
	dhCer(d18∶0/24∶1)	28.6	650.3	632.5	150	24	Cer(d17∶1/24∶1)
	dhCer(d18∶0/24∶0)	30.4	652.3	634.4	180	24	Cer(d17∶1/24∶1)
**LC-MS/MS run 3: For sphingomyelines**							
1	Lyso-SM(d18∶1)	2.0	464.8	170	183.9	24	Lyso-SM(d17∶1)
	Lyso-SM(d17∶1)	2.0	450.8	160	183.9	24	ISTD
	SM(d18∶1/2∶0)	3.6	507.3	183.9	160	24	SM(d18∶1/17∶0)
	SM(d18∶1/6∶0)	4.9	563.3	183.9	100	24	SM(d18∶1/17∶0)
2	SM(d18∶1/12∶0)	7.5	647.4	183.9	180	33	SM(d18∶1/17∶0)
	SM(d18∶1/16∶0)	9.0	703.5	184	160	30	SM(d18∶1/17∶0)
	SM(d18∶1/18∶1)	9.3	729.4	183.9	190	33	SM(d18∶1/17∶0)
	SM(d18∶1/17∶0)	9.4	717.5	184	100	30	ISTD
	SM(d18∶1/18∶0)	9.8	731.6	183.9	190	33	SM(d18∶1/17∶0)
	SM(d18∶1/24∶1)	11.9	813.6	183.9	240	30	SM(d18∶1/17∶0)
	SM(d18∶1/24∶0)	13.0	815.6	183.9	200	33	SM(d18∶1/17∶0)

Seg, segment; RT, retention time; Pre-ion, precursor ion; Pro-ion, product ion; CE, collision energy; ISTD, internal standard.

* Cer(d18∶1/26∶1) and Cer(d18∶1/26∶0) were quantitated using the calibration curve of the closest counterpart when commercial standards were not available. Cer(d18∶1/26∶1) ∼Cer(d18∶1/24∶1) and Cer(d18∶1/26∶0) ∼ Cer(d18∶1/24∶0).

### 2.5 Quantitation of Protein Content

The protein content of tissue homogenates was determined with the Bio-Rad Protein Assay using BSA as a reference standard [32].

### 2.6 Statistical Analysis

Significant differences between the two groups were evaluated by the Mann-Whitney U test. The data (p≤0.05) was then exported to SIMCA-P+12.0.1 (Umetrics AB, Sweden) for multivariate data analysis. OPLS-DA was used for modeling the differences between all groups. The OPLS-DA model was generated using normalized, mean-centered and unit-variance scaled data from each group. The criterias for each potential biomarker were: 1. A variable importance in projection greater than one; 2. The jack-knife uncertainty bar excluded zero; 3. The absolute value of Pcorr in the S-plot was greater than 0.58 [33]. Correlations between the acquired potential biomarkers and indicators of DTH severity (ear swelling and splenic index) were evaluated by Spearman’s correlation analysis. Correlation evaluation and normality tests were performed using SPSS 18.0.

## Results and Discussion

### 3.1 DTH Model Verification and the Effects of Triptolide

#### 3.1.1 Verification of the DTH model

**Figure 2:**
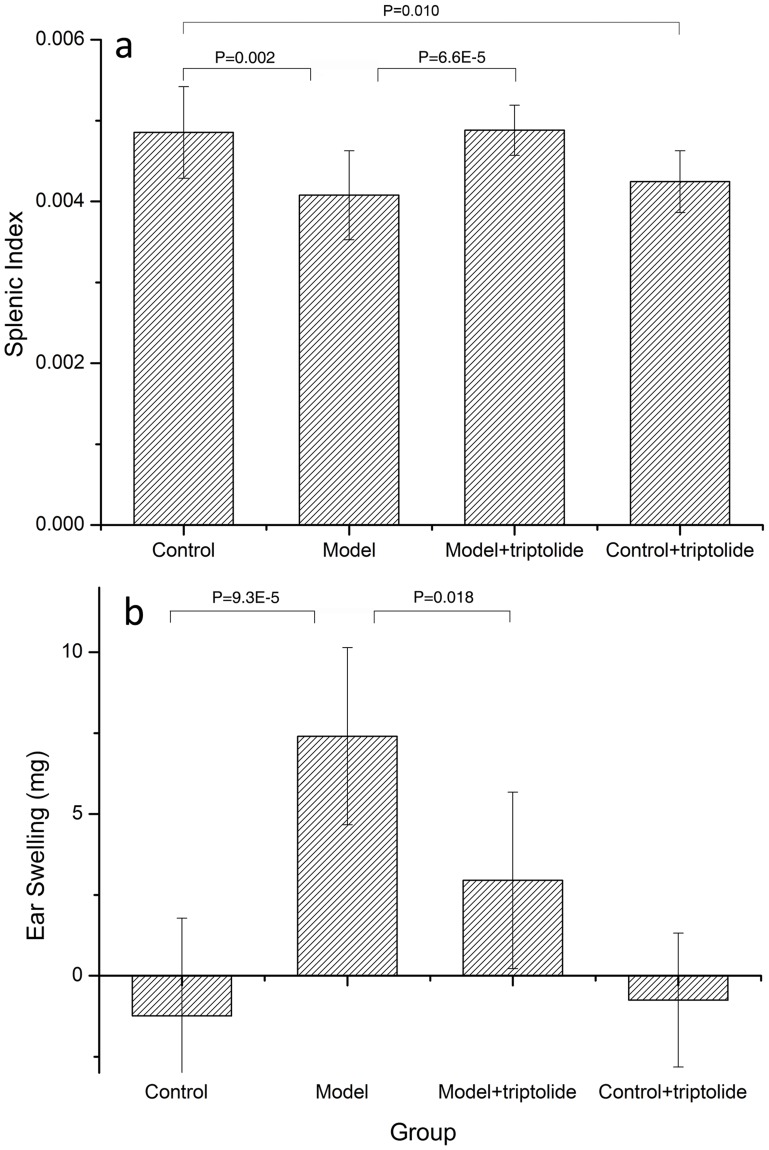
DTH model validation. a) The splenic index of each group. The splenic index is expressed as the ratio of the weight of the spleen weight to the weight of the mouse. The four groups include: control, DTH model, DTH model+triptolide and control+triptolide, each of which contains eight samples. Statistical differences detected between the control & DTH model groups and the DTH model & DTH model+triptolide groups are indicated by the P-value between each group. b) Ear swelling in each group. Ear swelling was expressed as the difference between the weight of left and right ear patches obtained from 8 mm punches 30 h after challenge. The punches were obtained in a blinded manner. Data are expressed as the mean ± SD, and the values for each sample are the mean of eight separate samples.

Tissue swelling at sites of stimuli contact is a symptom of autoimmune disease [34]. The relative weight of organs, such as the spleen, is also negatively associated with the severity of DTH diseases. Therefore, ear swelling and splenic index were used to estimate the severity of DTH [29], [35]. As expected, the average splenic index of mice was significantly lower in the DTH model group (–16%) than in the control group (P = 0.002, ***Figure 2a***). The average ear swelling in mice in the DTH model group treated with DNFB was significantly increased compared with the control group, which was treated only with vehicle (P = 9.3E-5, ***Figure 2b***). These measurements confirm that a model of DTH was successfully established in these mice.

#### 3.1.2 Effects of triptolide on DNFB-induced DTH model mice

The DNFB-induced DTH reaction is a Th1 cell-mediated pathologic response involving T cell activation and the production of numerous cytokines [36], [37]. We analyzed the effect of triptolide treatment on the DTH response in BALB/c mice. When administered to DTH model mice at a dosage of 70 µg/kg (i.p.), triptolide increased the splenic index by 19% (P = 6.6E-5, ***Figure 2a***) and suppressed ear swelling by 60% (P = 0.018, ***Figure 2b***) compared to mice in the DTH group. These findings confirm that treatment with 70 µg/kg triptolide has a therapeutic effect in DTH model mice.

### 3.2 Sphingolipidomic Analysis of DTH Mice before and Following Triptolide Treatment

Research published by Piali et al indicated that Sph (d18∶1)-1-P and its receptor are involved in inhibition of edema formation, inflammatory cell accumulation and cytokine release in the skin of mice with DTH [38]. Since sphingolipids function as a network [39], it was proposed that this network is responsible for mediating the development of DTH, and that triptolide plays a role in suppressing this process by regulating the metabolism of sphingolipids. This study highlights the importance of studying sphingolipids as a network rather than as independent biomolecules and examining the connection between inflammation and medication. Actually there is something interesting we have found from sphingolipidomic analysis of DTH and effects of triptolide. For the record, glucosylceramides and galactosylceramides share the same MRM transitions and do not resolve on C8 columns [27], so in the real biological sample analysis the peaks formerly labeled “glucosylceramide (GlcCer)” would be more accurately labeled "monohexosylceramide (HexCer)" to take into account the galactosylceramide component. Significant inference can be found on the MRM chromatograph of some target compounds whose mass transition pairs are 2 more than those of another compound (***Figure S3***). To avoid such inference separation on column is necessary for quantification. This HPLC-MS/MS method was validated according to the principles of Good Laboratory Practice and the Guidance of Industry Bioanalytical Method Validation [40], [41]. Validation result is shown in ***Table S1*** and ***Table S2.***

#### 3.2.1 Elevated sphingolipid levels in the spleens and livers of DTH mice are decreased by triptolide treatment

**Figure 3:**
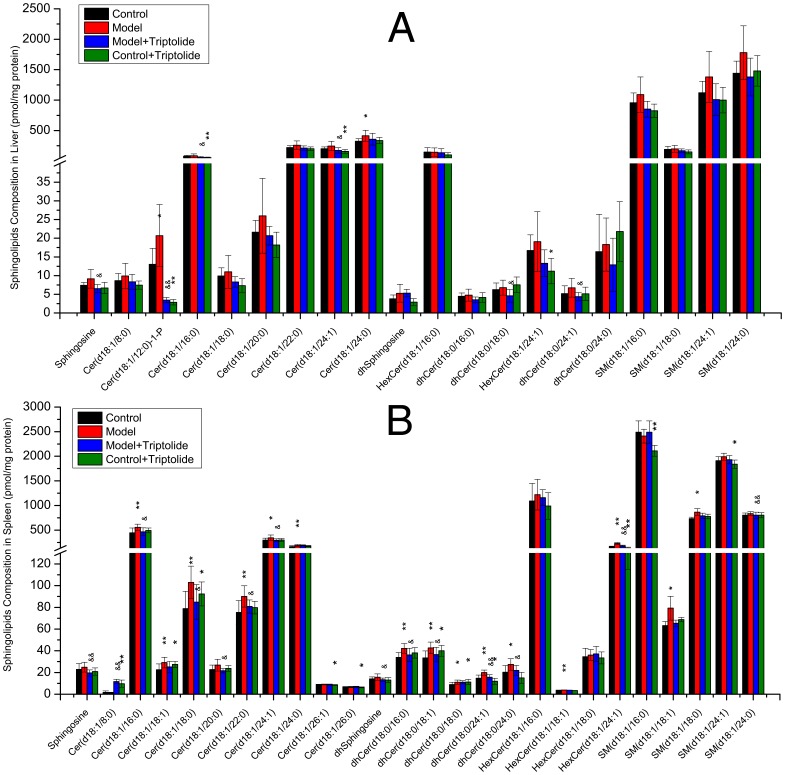
Sphingolipid composition of mice liver or spleen measured by triple quadruples MS/MS. Four groups including: control, model, model+triptolide and control+triptolide, each of which contains 8 samples. Sphingolipids were isolated from liver or spleen homogenate corresponding to 1 mg protein. Bars are expressed as means ± SD, values for each tissue sample are the average of 8 samples separately (pmol/mg protein). Statistical difference from control or model group is indicated with an asterisk or a “&”, respectively. * or &: p<0.05 and ** or &&: p<0.01.

Compositional analysis of common sphingolipids in the spleen and liver showed that the overall level of sphingolipids in DNFB-induced DTH mice was increased, especially Cer(d18∶1/12∶0)-1-P and Cer(d18∶1/24∶0) in the liver (P<0.05, ***Figure 3A***) and six ceramides, five dihydroceramides, two hexosylceramides and two sphingomyelins in the spleen (P<0.05, ***Figure 3B***). In mice treated with triptolide mitigated ear swelling and recovered splenic index were observed, which accompanied with a decrease in the overall level of sphingolipids, especially sphingosine, Cer(d18∶1/12∶0)-1-P, two ceramides and two dihydroceramides in the liver (P<0.05, ***Figure 3A***) and sphingosine, dhsphingosine, HexCer(d18∶1/24∶1), SM(d18∶1/24∶0), five ceramides, three dihydroceramides in the spleen (P<0.05, ***Figure 3B***).

**Figure 4:**
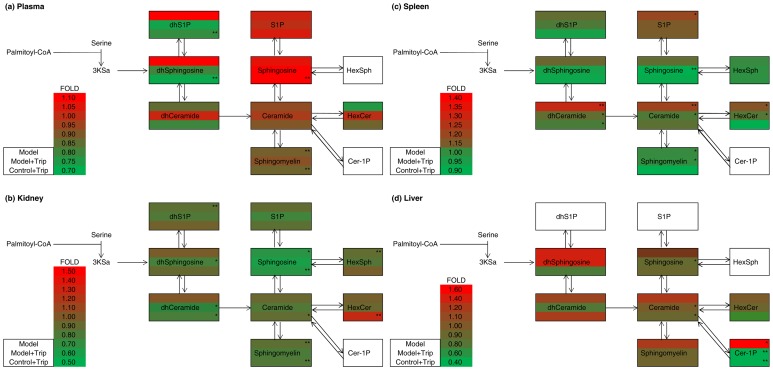
Visualization of sphingolipid metabolites in the plasma, kidney, spleen and liver. The figure depicts sphingolipid metabolites that participate in the early steps of sphingolipid biosynthesis. The pathway maps are overlaid by the fold changes in subclasses of sphingolipid metabolites between the control group and other groups by asterisks indicating the statistical significance on the right side (* = P<0.05, ** = P<0.01; n = 8). The content of each sphingolipid metabolite was determined as the mean of eight independent parallel samples.

The pathway heat map in ***Figure 4*** shows the fold changes in the each sub-class sphingolipid. With the exception of Cer-1-Ps, which were not detected, the levels of all other subclasses of sphingolipids were elevated in the spleens of mice in the DTH model group. In mice treated with triptolide, these elevations were suppressed. When control mice were treated with triptolide alone, the overall level of sphingolipids was decreased, indicative of the immunosuppressive effect of triptolide *(* ***Figure 4c*** *)*. In the liver, similar effects were observed in the levels of all sphingolipids except dhCeramide, which was elevated in the control+triptolide group. These results demonstrate that DTH increases the overall levels of sphingolipids in the liver and spleen, possibly by triggering the activation of serine palmitoyl transferase or ceramide synthase leading to the up-regulation of de novo sphingolipid synthesis, or sphingomyelinase leading to the up-regulation of ceramides. Treatment with triptolide prevented the increase in sphingolipid levels, possibly by mediating the inactivation of serine palmitoyl transferase or ceramide synthase.

**Figure 5:**
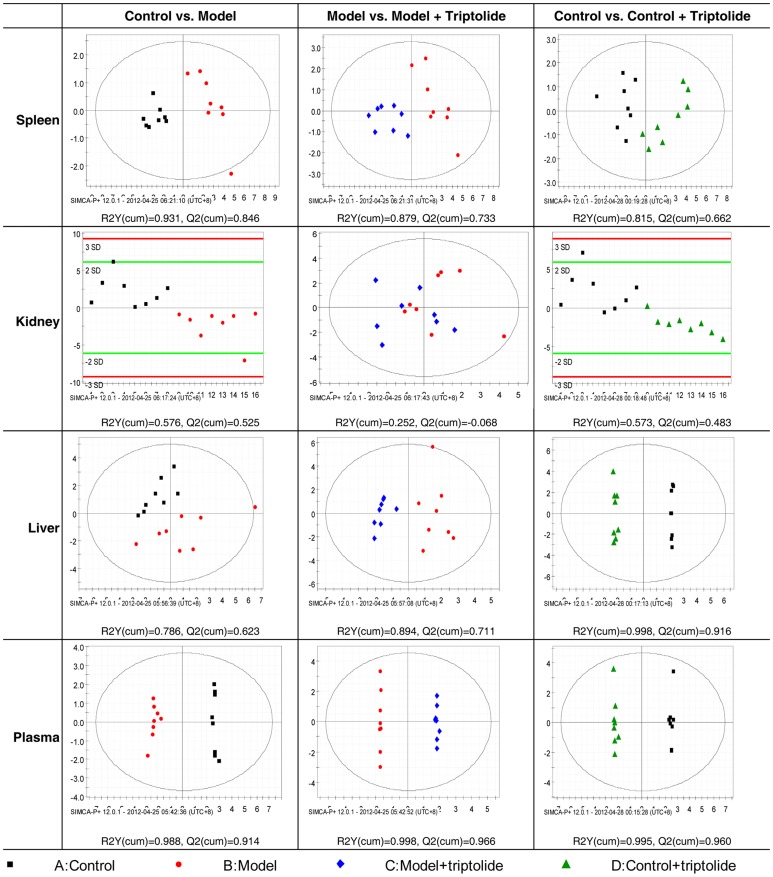
Score plots from supervised OPLS-DA. Analysis showed distinct clustering between each group in the spleen, kidney, liver and plasma. R2Y(cum) and Q2(cum) determined by Simca P+12.0.1 are shown under each panel and indicate the stability and predictability of the model.

To further study the subtle differences between the four groups, OPLS-DA was employed. Before analysis, variables that were not significantly different between the DTH model and control groups (p>0.05) were eliminated based on the results of the Mann-Whitney U test according to Lin et al [42]. Classification results are shown in ***Figure 5*** with R2Y(cum) and Q2(cum) indicated below each chart. R2Y(cum) is the fraction of the sum of squares of all Y-values explained by the current latent variables, and the Q2(cum) is the cumulative Q2 for the extracted latent variables. Distinct clustering between model group mice and control mice was achieved. In the values obtained from spleen samples, shown in the top row of ***Figure 5***, inflammation gradually recovers in mice treated with triptolide. Values from the model+triptolide group are on the left side of the panel and produced a pattern similar to that of the control group, indicating that these mice present a similar profile in sphingolipidomic analysis. Furthermore, OPLS-DA analysis suggested that both spleen and liver had ten but different sphingolipid potential biomarkers reflecting the DTH severity and response to triptolide as indicated in ***Table 2***.

**Table 2:** Sphingolipid biomarkers found in spleen, liver, plasma and kidney.

	Spleen	Liver	Plasma	Kidney
Sphingolipid	Cer(d18∶1/16∶0)	Cer(d18∶1/18∶0)	Cer(d18∶1/16∶0)	Cer(d18∶1/18∶0)
Biomarker	Cer(d18∶1/18∶0)	Cer(d18∶1/8∶0)	Cer(d18∶1/18∶0)	Cer(d18∶1/8∶0)
	Cer(d18∶1/18∶1)	dhCer(d18∶0/18∶1)	Cer(d18∶1/20∶0)	dhCer(d18∶0/18∶1)
	Cer(d18∶1/22∶0)	dhSphingosine-1-P	dhsphingosine	dhSphingosine-1-P
	Cer(d18∶1/24∶0)	HexCer(d18∶1/18∶0)	dhsphingosine-1-P	HexCer(d18∶1/18∶0)
	dhCer(d18∶0/16∶0)	SM(d18∶1/16∶0)	HexCer(d18∶1/16∶0)	SM(d18∶1/16∶0)
	dhCer(d18∶0/18∶1)	SM(d18∶1/18∶0)	HexCer(d18∶1/24∶1)	SM(d18∶1/18∶0)
	dhCer(d18∶0/24∶1)	SM(d18∶1/24∶0)	SM(d18∶1/16∶0)	SM(d18∶1/24∶0)
	HexCer(d18∶1/24∶1)	SM(d18∶1/24∶1)	SM(d18∶1/18∶0)	SM(d18∶1/24∶1)
	SM(d18∶1/18∶1)	Sphingosine	SM(d18∶1/18∶1)	Sphingosine
			SM(d18∶1/24∶1)	
			Sphingosine	

#### 3.2.2 Triptolide treatment prevented the decrease in plasma sphingolipid levels but enhanced the decrease in sphingolipid levels in the kidneys of DTH mice

**Figure 6:**
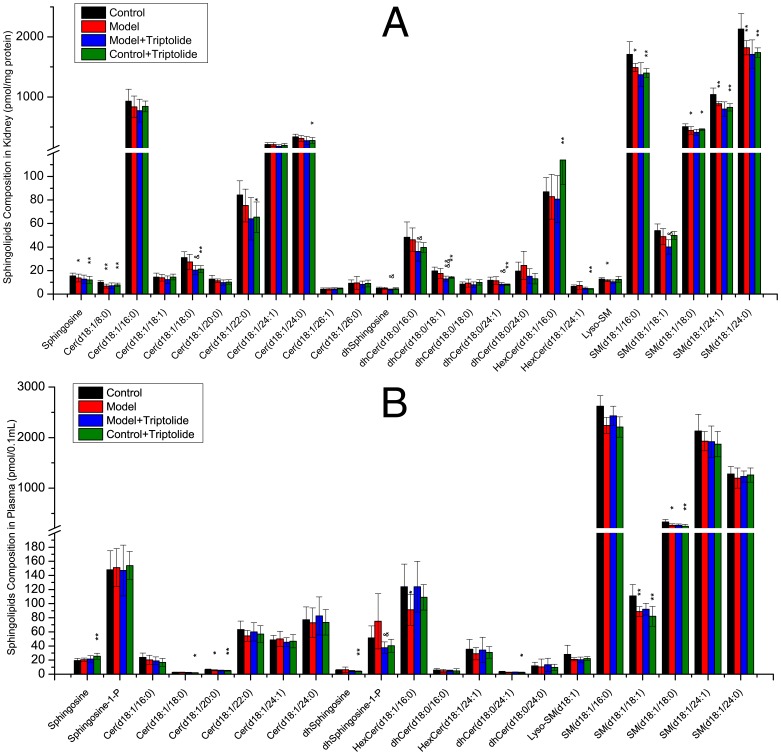
Sphingolipid composition of mice kidney or plasma measured by triple quadruples MS/MS. Four groups including: control, model, model+triptolide and control+triptolide, each of which contains 8 samples. Sphingolipids were isolated from kidney homogenate corresponding to 1 mg protein or from 0.1 mL plasma. Bars are expressed as means ± SD, values for each sample are the average of 8 samples separately (pmol/mg protein, 0.1 mL plasma). Statistical difference from control or model group is indicated with an asterisk or a “&”, respectively. * or &: p<0.05 and ** or &&: p<0.01.

Compositional analysis of common sphingolipids in the kidneys and plasma of DTH model mice revealed a reduction in most sphingolipids, especially sphingosine, Cer(d18∶1/8∶0) and five sphingomyelins in the kidneys (P<0.05, ***Figure 6A***) and Cer(d18∶1/20∶0), HexCer(d18∶1/16∶0), SM(d18∶1/18∶1) and SM(d18∶1/18∶0) in plasma (P<0.05, ***Figure 6B***).

In mice treated with triptolide, the overall level of sphingolipids in plasma was elevated slightly corresponding to the mitigated ear swelling and recovered splenic index (***Figure 6B***). However, mice treated with triptolide alone entered an abnormal sphingolipid-metabolic state. For example, sphingosine levels in plasma were elevated markedly compared with the control group (P<0.01, ***Figure 6B***), while Cer(d18∶1/18∶0), Cer(d18∶1/20∶0), dhsphingosine, dhCer(d18∶0/24∶1), SM(d18∶1/18∶1) and SM(d18∶1/18∶0) were markedly decreased compared with the control group (P<0.05, ***Figure 6B***).

As shown in ***Figure 4a*** *,* the fold change in each sphingolipid sub-class in plasma showed that dhsphingosine-1-P, dhsphingosine, sphingosine-1-P and sphingosine were up-regulated in the DTH model group compared with the control group, while N-acyl sphingolipids such as dhceramide, ceramide, HexCer and sphingomyelin were down-regulated. In mice treated with triptolide, N-acyl sphingolipids were up-regulated, while dhsphingosine and dhS1P were down-regulated, which might result from the induction of ceramide synthase by triptolide. However, control mice treated with triptolide alone did not exhibit this response; conversely, overall levels of sphingolipids were down-regulated, except sphingosine and S1P, which were up-regulated. Since sphingolipid synthesis is a dynamic and a multi-dimensional pathway, the regulation steps on the sphingolipid synthetic pathway by triptolide requires further study by checking the enzyme activities.

Sphingolipidomic analysis of the kidney produced some unexpected findings. While DTH resulted in a marked decrease in the overall level of sphingolipids, treatment with triptolide reduced sphingolipid levels further. Kidneys of mice treated with triptolide alone also showed marked down-regulation of sphingolipid levels (P<0.05**,** ***Figure 4b***). These results indicated that the kidneys in model mice treated with triptolide continued to deteriorate, even when other organs were recovering.

In previously studies, DTH and improper dose of triptolide could cause significant kidney dysfunction [16], [43], [44]. Thus, as described above in this study, the down-regulation of sphingolipid levels might correlate with the exacerbation of kidney dysfunction. The pathway heat map shown in ***Figure 4b*** outlines this relationship. Thus, both DTH induced by DNFB and treatment with triptolide have harmful effects on the kidney. Many previous studies have shown the deleterious effects of triptolide on kidney function; TWHF was toxic to rodent kidneys even at normal dosage levels [15], [16], [44]–[46]. These early studies reported that oxidative stress caused by triptolide is involved in drug-induced nephrotoxicity by reducing the activity of renal superoxide dismutase and glutathione peroxidase and increasing renal malondialdehyde content [16], [44]. However, although the accumulation of HexCer(d18∶1/16∶0) might be caused by the degradation of complex glycosphingolipids induced by triptolide, no connection between the nephrotoxic effects of triptolide and its effects on sphingolipid levels has been reported so far.

The OPLS-DA charts from plasma and kidney *(* ***Figure 5*** *)* samples showed that all comparisons produced acceptable R2Y(cum) and Q2(cum) values, indicating that the model possessed good stability and predictability, respectively. However, stability and predictability were poor for the comparison of kidney samples between the DTH model and DTH model+triptolide groups. Thus, no potential biomarker was identified by OPLS-DA analysis of these kidney samples, suggesting that the effect of triptolide on the kidney of the DTH model mice was not so evident. Finally, several points needed to be pointed out were the identical alterations of sphingolipids in plasma. The R2Y(cum) and Q2(cum) values produced by the comparison of plasma sphingolipid levels were greater than 0.9, better than those in other tissues. As a result, the potential biomarkers identified in plasma are most likely to represent accurate biomarkers than those identified in other tissues. In addition, samples from plasma or blood are easier to obtain than samples from organs. These results have great significance for clinical applications, including the diagnosis and treatment of DTH diseases.

Based on the criteria for the identification of potential biomarkers, OPLS-DA analysis detected several potential biomarkers of DTH and its response to triptolide, including twelve and ten sphingolipids in plasma and kidneys, respectively, as indicated in ***Table 2*** ***.***

### 3.3 Sphingolipids Correlated with Ear Swelling and Splenic Index

**Figure 7:**
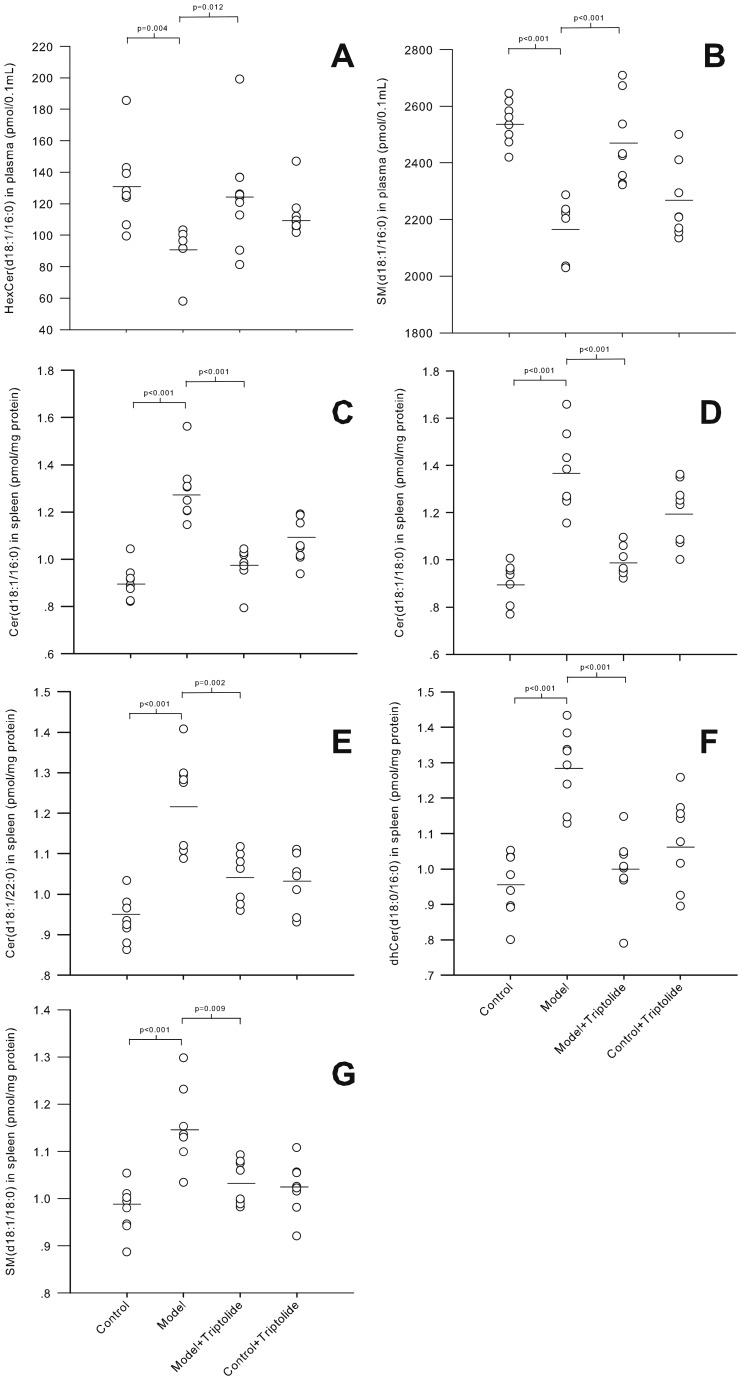
Sphingolipid biomarkers of DTH disease severity and response to triptolide treatment. The levels of seven potential sphingolipid biomarkers in the plasma (A, B) and spleen (C-G) in control, DTH model, DTH model+triptolide and control+triptolide groups. The sphingolipids were identified based on data obtained by OPLS-DA with a significant correlation (p<0.05) to ear swelling and splenic index as indicators of DTH severity. Significant differences between the two groups were evaluated by Mann-Whitney U test with the P-value shown above each plot.

During model validation, ear swelling and splenic index were employed to establish the severity of DTH in model mice. Seven of the sphingolipid biomarkers identified above correlated significantly with the degree of ear swelling and the splenic index, as shown in ***Table 3*** ***.*** These biomarkers were able to discern DTH severity; their levels were markedly different between control and DTH model groups and inverse alterations following successful treatment with triptolide (***Figure 7***). Thus, these sphingolipid biomarkers may be able to assist the evaluation of DTH diseases.

**Table 3:** Correlation of sphingolipid biomarkers with ear swelling and splenic index.

		P-value		R-value	
Tissue	Sphingolipids	Ear swelling	Splenic index	Ear Swelling	Splenic index
Plasma	GluCer(d18∶1/16∶0)	0.0215	0.0324	0.29	0.34
Plasma	SM(d18∶1/16∶0)	0.0509	0.0016	0.32	0.43
Spleen	Cer(d18∶1/16∶0)	0.0345	0.0008	0.35	0.45
Spleen	Cer(d18∶1/18∶0)	0.0487	0.0002	0.32	0.54
Spleen	Cer(d18∶1/22∶0)	0.0131	0.0007	0.46	0.52
Spleen	dhCer(d18∶0/16∶0)	0.0633	0.0017	0.36	0.41
Spleen	SM(d18∶1/18∶0)	0.0358	0.0001	0.32	0.55

This correlation between DTH severity and biomarker levels was observed in plasma and the spleen, but not in the kidney or liver. This is expected in the kidney due to the nephrotoxic effects of triptolide. The liver plays a major role in metabolism, and is not as closely linked with the immune system as other organs, such as the spleen, lymph nodes, thymus and kidneys. Thus, the sphingolipids in the liver may not be associated with DTH or other immunological diseases.

### 3.4 Effects of Triptolide on Control Mice

Although triptolide has therapeutic benefits in patients with autoimmune and inflammatory diseases, its toxic potential cannot be ignored [47]. In this study, the upper limit of therapeutic triptolide (70 µg/kg) was administered to healthy mice. As shown in ***Figure 2a***, the splenic index was decreased markedly in the control+triptolide group compared to the control group, reflecting the toxicity of triptolide. Ear swelling was not considered significant as it is one of the characteristic responses of DNFB-induced DTH and not a response to triptolide.

Many sphingolipids exhibited significant variation in the control+triptolide group compared with the control group (***Figure 3*** ***,*** ***6***). In fact, the OPLS-DA plots showed that the control+triptolide mice (green triangles) were distributed separately from the control group (black dots; ***Figure 5***).

In summary, this study examined the toxicity of triptolide from a sphingolipidomic perspective, which might contribute to a better understanding of the mechanism of triptolide toxicity.

### Conclusion

In this study, feasible, accurate and robust LC-MS/MS methods were developed for the quantification of 43 core sphingolipids covering the core network of sphingolipid metabolism in biological samples. In addition to ordinary shotgun metabolomics methods, targeted “-omic” methods employing LC coupled with triple quadrupole mass spectrometry and analog internal standards could offer incomparable accuracy and sensitivity. Moreover, targeted sphingolipidomic analysis followed by multivariate analysis could become a new strategy for the identification of biomarkers in many kinds of biological samples.

The use of DTH model mice treated with triptolide uncovered the pathogenesis of DTH diseases and the effect of triptolide treatment in three different organs and plasma. In addition, 23 potential biomarkers were identified by OPLS-DA. Up-regulation of the overall level of sphingolipids was observed in the spleen and liver in DTH model mice, while down-regulation was observed in the plasma and kidneys, which means that treatment with DTH should be taken separately and more target among the body. Triptolide treatment prevented DTH-induced alterations in sphingolipid levels in the plasma, liver and spleen, indicating that the pharmacological modification of sphingolipid levels may be a novel therapeutic method for the treatment of patients with DTH diseases. Furthermore, seven sphingolipid biomarkers were identified that correlated markedly with the severity of DTH, indicating that the development of DTH and the effectiveness of treatment with triptolide were the result of alterations in sphingolipid levels. On the other hand, triptolide treatment enhanced the down-regulation of sphingolipids in kidney, which underscores the nephrotoxic effects of triptolide. The levels of sphingolipids in the kidneys might be useful as a characteristic index for the identification or evaluation of nephrotoxic lesions or as a novel therapeutic target for the treatment of nephrotoxicity caused by external stimuli.

## Supporting Information

Figure S1 **Representative MS/MS fragmentation patterns of initial positive molecular ions, generated upon ESI source.**
(TIF)

Figure S2 **Chromatographic analysis of samples at the lowest limit of quantification and mice spleen samples (∼1 mg protein) to determine the specificity of each method.** For the Cer/Cer-1-P/Sph/S1P method, the analytes are listed according to the number above the peak: 1, Sph(d17∶1); 2, Sph(d18∶1); 3, Sph(d17∶1)-1-P; 4, Sph(d18∶1)-1-P; 5, Cer(d18∶1/2∶0); 6, Cer(d18∶1/2∶0)-1-P; 7, Cer(d18∶1/4∶0); 8, Cer(d18∶1/6∶0); 9, Cer(d18∶1/8∶0); 10, Cer(d18∶1/8∶0)-1-P; 11, Cer(d18∶1/10∶0); 12, Cer(d18∶1/12∶0); 13, Cer(d18∶1/12∶0)-1-P; 14, Cer(d18∶1/14∶0); 15, Cer(d18∶1/16∶0); 16, Cer(d18∶1/18∶1); 17, Cer(d17∶1/18∶0); 18, Cer(d18∶1/18∶0); 19, Cer(d18∶1/16∶0)-1-P; 20, Cer(d18∶1/18∶1)-1-P; 21, Cer(d18∶1/20∶0); 22, Cer(d17∶1/24∶1); 23, Cer(d18∶1/22∶0); 24, Cer(d18∶1/24∶1); 25, Cer(d18∶1/24∶0). For dhSPL/HexSPL the numbers correspond to: 1, HexSph(d18∶1); 2, dhSph(d17∶0); 3, dhSph(d18∶0); 4, dhS1P(d17∶1); 5, dhS1P(d18∶1); 6, dhCer(d18∶0/2∶0); 7, GlcCer(d18∶1/8∶0); 8, dhCer(d18∶0/6∶0); 9, dhCer(d18∶0/8∶0); 10, HexCer(d18∶1/12∶0); 11, HexCer(d18∶1/16∶0); 12, HexCer(d18∶1/18∶1); 13, HexCer(d18∶1/18∶0); 14, dhCer(d18∶0/16∶0); 15, Cer(d17∶1/18∶0); 16, dhCer(d18∶0/18∶1); 17, dhCer(d18∶0/18∶0); 18, HexCer(d18∶1/24∶1); 19, Cer(d17∶1/24∶1); 20, dhCer(d18∶0/24∶1); 21, dhCer(d18∶0/24∶0). For SM, the numbers correspond to: 1, Lyso-SM(d17∶1); 2, Lyso-SM(d18∶1); 3, SM(d18∶1/2∶0); 4, SM(d18∶1/6∶0); 5, SM(d18∶1/12∶0); 6, SM(d18∶1/16∶0); 7, SM(d18∶1/18∶1); 8, SM(d18∶1/17∶0); 9, SM(d18∶1/18∶0); 10, SM(d18∶1/24∶1); 11, SM(d18∶1/24∶0).
(TIF)

Figure S3 **Significant inference (emphasized in grey) can be seen on the MRM chromatographs.** Some target compounds whose mass transition pairs are 2 Da more than those of another compound will result in such inference. Avoidance of them separation is necessary for quantification.
(TIF)

Table S1 **Validation results for precision, accuracy and absolute recovery.**
(DOCX)

Table S2 **Validation results for stock solution stability.**
(DOCX)

Information S1 Sample preparation: Procedures of sample preparation before HPLC-MS/MS. Standard curves and quality control samples: Preparation procedures of standard curve of each target compound and the quality control samples. Gradient elution programs: Gradient elution programs for each subclasses of sphingolipids. Method development: Key points about the development of this quantitative sphingolipidomic platform. They answered those following questions: 1) How were the chromatography and mass spectrometry conditions optimized? 2) Why we employed the segmental multiple reaction monitoring method on quantification of sphingolipids and what was the advantages? 3) How were the extraction method optimized? 4) What the usage of the unnatural sphingolipids detected by this platform? Method Validation: Procedures and results of the HPLC-MS/MS method validation.
(DOCX)
