# Supplementary figures and images for: Sphingolipids as New Biomarkers for Assessment of Delayed-Type Hypersensitivity and Response to Triptolide
Source: PLoS One. 2012 Dec 26;7(12):e52454. doi: 10.1371/journal.pone.0052454 (PMC3530451; doi:10.1371/journal.pone.0052454)

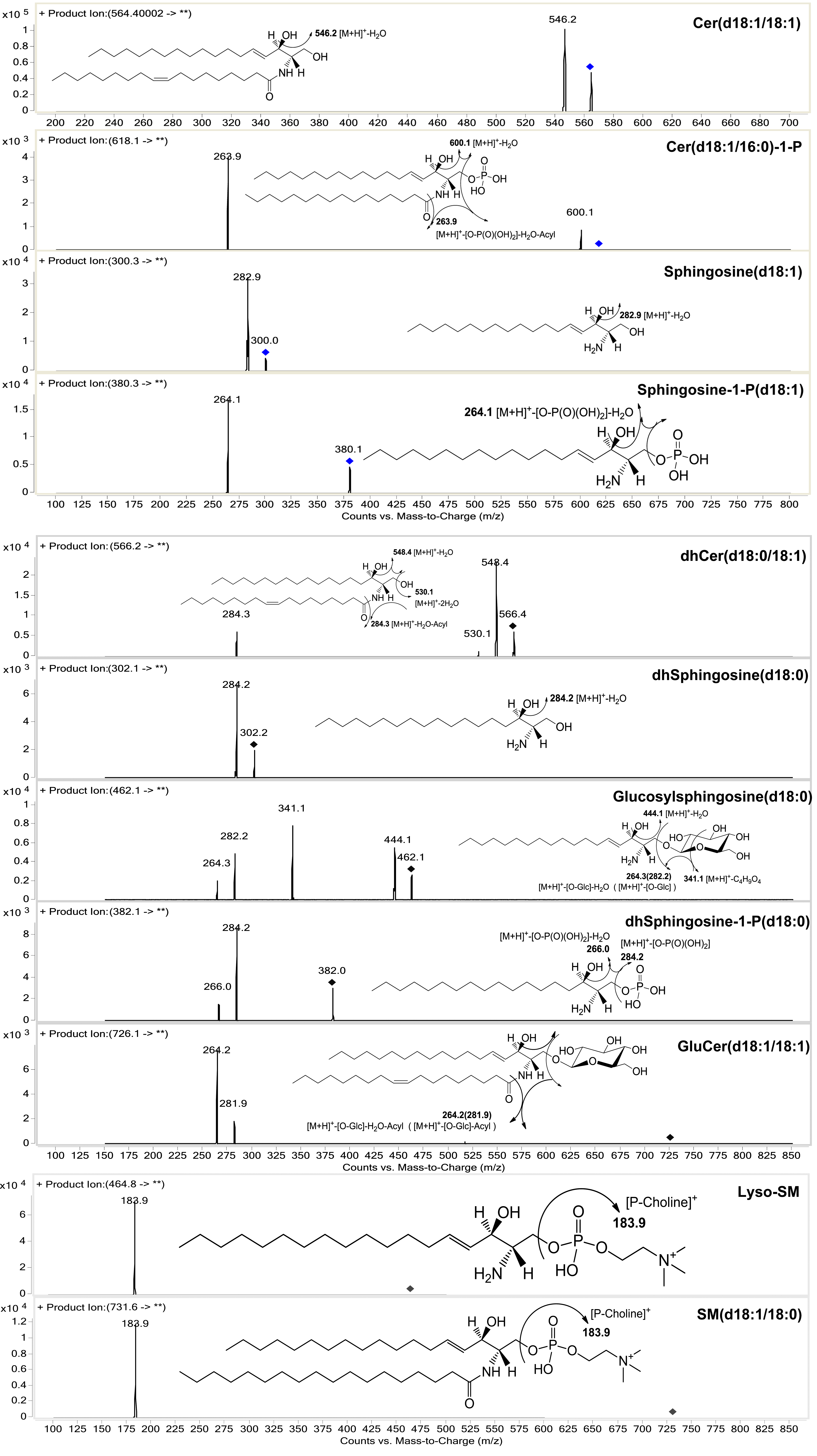

Supplement: Figure S1 — Representative MS/MS fragmentation patterns of initial positive molecular ions, generated upon ESI source. (TIF) [file pone.0052454.s001.tif]

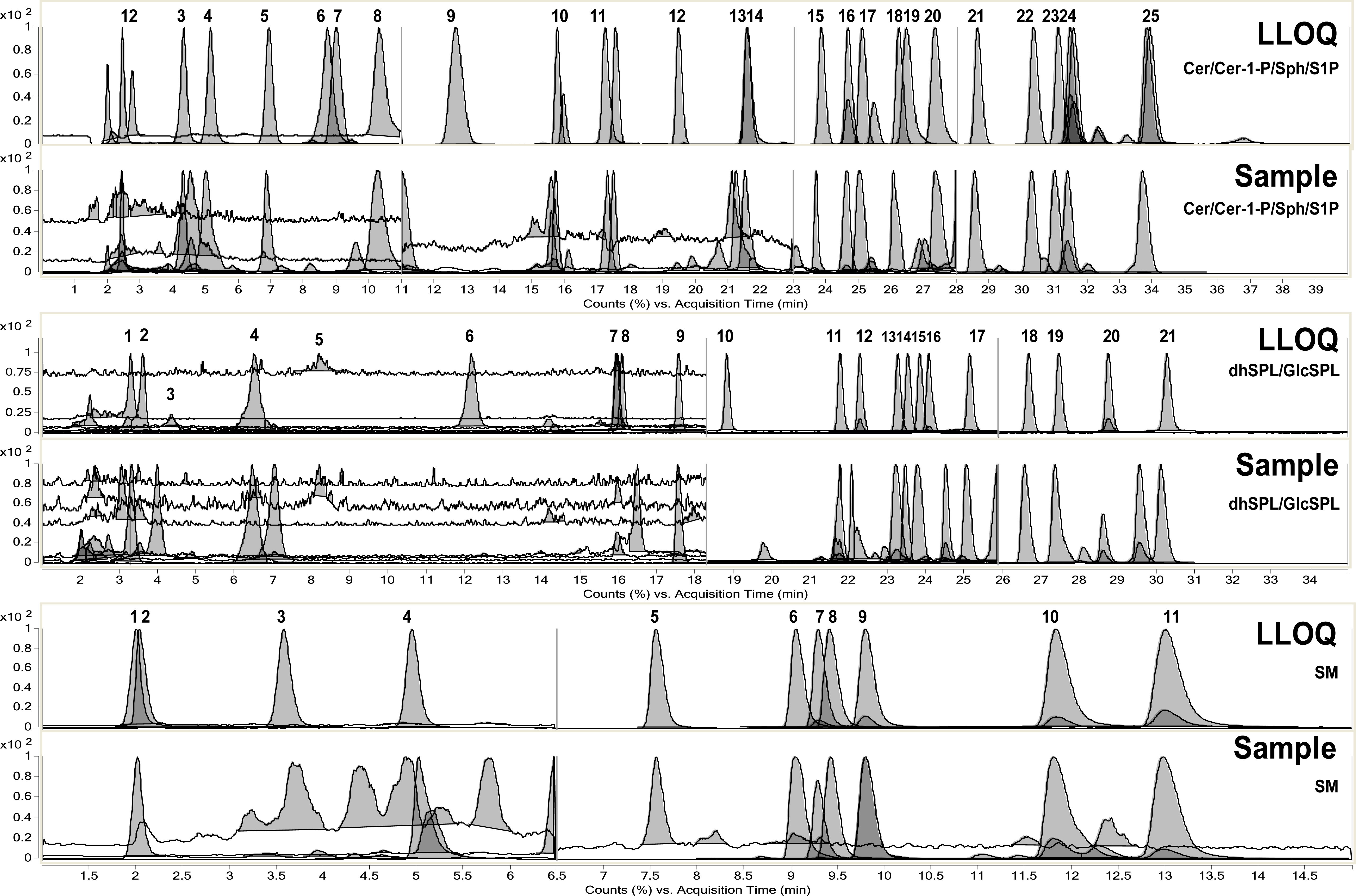

Supplement: Figure S2 — Chromatographic analysis of samples at the lowest limit of quantification and mice spleen samples (∼1 mg protein) to determine the specificity of each method. For the Cer/Cer-1-P/Sph/S1P method, the analytes are listed according to the number above the peak: 1, Sph(d17∶1); 2, Sph(d18∶1); 3, Sph(d17∶1)-1-P; 4, Sph(d18∶1)-1-P; 5, Cer(d18∶1/2∶0); 6, Cer(d18∶1/2∶0)-1-P; 7, Cer(d18∶1/4∶0); 8, Cer(d18∶1/6∶0); 9, Cer(d18∶1/8∶0); 10, Cer(d18∶1/8∶0)-1-P; 11, Cer(d18∶1/10∶0); 12, Cer(d18∶1/12∶0); 13, Cer(d18∶1/12∶0)-1-P; 14, Cer(d18∶1/14∶0); 15, Cer(d18∶1/16∶0); 16, Cer(d18∶1/18∶1); 17, Cer(d17∶1/18∶0); 18, Cer(d18∶1/18∶0); 19, Cer(d18∶1/16∶0)-1-P; 20, Cer(d18∶1/18∶1)-1-P; 21, Cer(d18∶1/20∶0); 22, Cer(d17∶1/24∶1); 23, Cer(d18∶1/22∶0); 24, Cer(d18∶1/24∶1); 25, Cer(d18∶1/24∶0). For dhSPL/HexSPL the numbers correspond to: 1, HexSph(d18∶1); 2, dhSph(d17∶0); 3, dhSph(d18∶0); 4, dhS1P(d17∶1); 5, dhS1P(d18∶1); 6, dhCer(d18∶0/2∶0); 7, GlcCer(d18∶1/8∶0); 8, dhCer(d18∶0/6∶0); 9, dhCer(d18∶0/8∶0); 10, HexCer(d18∶1/12∶0); 11, HexCer(d18∶1/16∶0); 12, HexCer(d18∶1/18∶1); 13, HexCer(d18∶1/18∶0); 14, dhCer(d18∶0/16∶0); 15, Cer(d17∶1/18∶0); 16, dhCer(d18∶0/18∶1); 17, dhCer(d18∶0/18∶0); 18, HexCer(d18∶1/24∶1); 19, Cer(d17∶1/24∶1); 20, dhCer(d18∶0/24∶1); 21, dhCer(d18∶0/24∶0). For SM, the numbers correspond to: 1, Lyso-SM(d17∶1); 2, Lyso-SM(d18∶1); 3, SM(d18∶1/2∶0); 4, SM(d18∶1/6∶0); 5, SM(d18∶1/12∶0); 6, SM(d18∶1/16∶0); 7, SM(d18∶1/18∶1); 8, SM(d18∶1/17∶0); 9, SM(d18∶1/18∶0); 10, SM(d18∶1/24∶1); 11, SM(d18∶1/24∶0). (TIF) [file pone.0052454.s002.tif]

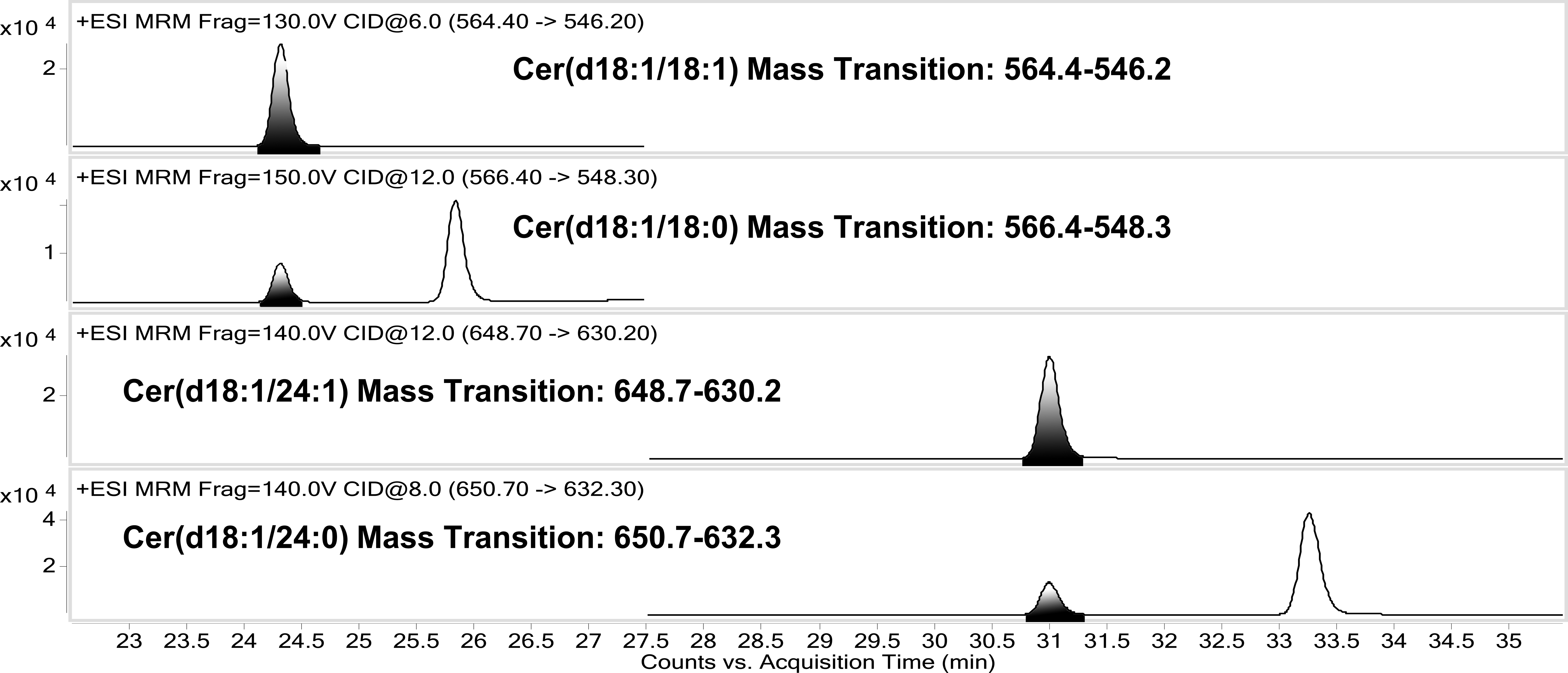

Supplement: Figure S3 — Significant inference (emphasized in grey) can be seen on the MRM chromatographs. Some target compounds whose mass transition pairs are 2 Da more than those of another compound will result in such inference. Avoidance of them separation is necessary for quantification. (TIF) [file pone.0052454.s003.tif]
