# Supplementary material for: Sphingolipids as New Biomarkers for Assessment of Delayed-Type Hypersensitivity and Response to Triptolide
Source: PLoS One. 2012 Dec 26;7(12):e52454. doi: 10.1371/journal.pone.0052454 (PMC3530451; doi:10.1371/journal.pone.0052454)
Supplement: Table S1 — Validation results for precision, accuracy and absolute recovery. (DOCX) [file pone.0052454.s004.docx]

**Table S1.** Validation results for precision, accuracy and absolute recovery

|  |  |  | **IA Pre** | **IE Pre** | **Accuracy** | **A-Reco** |
| --- | --- | --- | --- | --- | --- | --- |
| **Compound** | **r** | **Level** | [CV %] n=6 | | [%] n=6 | [%] |
| Sphingosine | 0.9927 | 1 | 4.3 | 12 | 103 | 73.3 |
|  |  | 2 | 10 | 11 | 93.3 | 76.1 |
|  |  | 3 | 3.5 | 9.2 | 95.4 | 72.8 |
|  |  | 4 | 4.6 | 9.6 | 102 | 71.4 |
| Cer(d18:1/4:0) | 0.9957 | 1 | 9.8 | 7.7 | 111 | 71.7 |
|  |  | 2 | 6.8 | 7.3 | 107 | 78.5 |
|  |  | 3 | 2.2 | 2.4 | 104 | 72.0 |
|  |  | 4 | 3.0 | 3.8 | 101 | 66.7 |
| Cer(d18:1/6:0) | 0.9924 | 1 | 11 | 7.7 | 84.7 | 71.4 |
|  |  | 2 | 9.1 | 9.5 | 98.4 | 75.2 |
|  |  | 3 | 4.2 | 6.4 | 113 | 72.2 |
|  |  | 4 | 12 | 13 | 102 | 72.1 |
| Cer(d18:1/8:0) | 0.9917 | 1 | 14 | 15 | 92.3 | 78.2 |
|  |  | 2 | 5.4 | 5.8 | 100 | 79.8 |
|  |  | 3 | 7.4 | 12 | 111 | 73.8 |
|  |  | 4 | 7.0 | 7.0 | 100 | 67.7 |
| Cer(d18:1/12:0) | 0.9916 | 1 | 7.6 | 8.8 | 116 | 71.3 |
|  |  | 2 | 7.7 | 7.3 | 108 | 74.7 |
|  |  | 3 | 5.0 | 4.7 | 111 | 77.0 |
|  |  | 4 | 7.7 | 7.1 | 97.7 | 66.2 |
| Cer(d18:1/14:0) | 0.9894 | 1 | 5.8 | 12 | 106 | 71.7 |
|  |  | 2 | 10 | 6.8 | 102 | 70.2 |
|  |  | 3 | 4.1 | 6.2 | 113 | 76.0 |
|  |  | 4 | 10 | 8.5 | 111 | 68.7 |
| Cer(d18:1/16:0) | 0.9957 | 1 | 14 | 5.4 | 102 | 76.8 |
|  |  | 2 | 4.5 | 7.5 | 101 | 71.0 |
|  |  | 3 | 7.7 | 11 | 102 | 77.2 |
|  |  | 4 | 3.3 | 3.2 | 99.7 | 69.9 |
| Cer(d18:1/18:1) | 0.9975 | 1 | 4.8 | 3.4 | 118 | 77.4 |
|  |  | 2 | 8.9 | 8.6 | 93.4 | 75.5 |
|  |  | 3 | 7.2 | 7.2 | 83.2 | 74.4 |
|  |  | 4 | 4.9 | 10 | 111 | 65.9 |
| Cer(d18:1/18:0) | 0.9973 | 1 | 7.0 | 4.0 | 114 | 79.0 |
|  |  | 2 | 2.8 | 2.9 | 105 | 78.8 |
|  |  | 3 | 2.0 | 1.9 | 103 | 74.4 |
|  |  | 4 | 4.9 | 5.4 | 94.2 | 70.4 |
| Cer(d18:1/20:0) | 0.9967 | 1 | 3.6 | 6.4 | 118 | 70.4 |
|  |  | 2 | 2.6 | 2.5 | 99.5 | 77.2 |
|  |  | 3 | 6.2 | 5.9 | 90.7 | 74.7 |
|  |  | 4 | 7.0 | 7.0 | 86.5 | 67.2 |
| Cer(d18:1/22:0) | 0.9965 | 1 | 4.1 | 8.5 | 121 | 78.3 |
|  |  | 2 | 3.5 | 3.4 | 111 | 77.8 |
|  |  | 3 | 4.8 | 4.2 | 95.0 | 70.2 |
|  |  | 4 | 10 | 9.9 | 86.0 | 69.9 |
| Cer(d18:1/24:1) | 0.9977 | 1 | 6.0 | 11 | 119 | 70.2 |
|  |  | 2 | 2.8 | 2.7 | 104 | 77.6 |
|  |  | 3 | 1.1 | 1.1 | 97.4 | 74.9 |
|  |  | 4 | 6.0 | 5.8 | 92.5 | 73.8 |
| Cer(d18:1/24:0) | 0.9966 | 1 | 7.7 | 8.7 | 106 | 72.4 |
|  |  | 2 | 3.3 | 4.1 | 116 | 70.1 |
|  |  | 3 | 6.0 | 5.4 | 102 | 73.9 |
|  |  | 4 | 11 | 8.4 | 84.1 | 68.5 |
| Sphingosine-1-P | 0.9934 | 1 | 7.1 | 9.9 | 89.0 | 72.2 |
|  |  | 2 | 7.4 | 8.8 | 108 | 75.7 |
|  |  | 3 | 5.2 | 3.2 | 106 | 71.6 |
|  |  | 4 | 4.3 | 3.8 | 103 | 67.9 |
| Cer(d18:1/2:0)-1-P | 0.9918 | 1 | 9.6 | 10 | 92.0 | 74.4 |
|  |  | 2 | 8.2 | 10 | 95.2 | 79.4 |
|  |  | 3 | 3.0 | 9.8 | 115 | 75.2 |
|  |  | 4 | 12 | 12 | 98.4 | 66.0 |
| Cer(d18:1/12:0)-1-P | 0.9908 | 1 | 5.2 | 8.5 | 116 | 70.5 |
|  |  | 2 | 13 | 3.6 | 95.8 | 80.7 |
|  |  | 3 | 13 | 3.7 | 86.8 | 75.8 |
|  |  | 4 | 12 | 5.3 | 104 | 72.4 |
| Cer(d18:1/16:0)-1-P | 0.9914 | 1 | 13 | 8.7 | 111 | 72.7 |
|  |  | 2 | 10 | 4.4 | 89.0 | 78.1 |
|  |  | 3 | 5.0 | 9.5 | 112 | 71.9 |
|  |  | 4 | 11 | 6.2 | 108 | 68.5 |
| Cer(d18:1/18:1)-1-P | 0.9974 | 1 | 11 | 8.6 | 108 | 71.8 |
|  |  | 2 | 6.1 | 14 | 99.9 | 77.3 |
|  |  | 3 | 9.6 | 14 | 106 | 76.9 |
|  |  | 4 | 11 | 12 | 103 | 67.7 |
| dhSphingosine | 0.9921 | 1 | 9.2 | 7.8 | 99.6 | 69.2 |
|  |  | 2 | 5.9 | 5.4 | 96.9 | 79.2 |
|  |  | 3 | 9.5 | 8.8 | 99.5 | 70.8 |
|  |  | 4 | 8.8 | 6.7 | 100 | 75.2 |
| dhCer(d18:0/2:0) | 0.9964 | 1 | 3.9 | 11 | 113 | 80.5 |
|  |  | 2 | 4.7 | 3.5 | 97.6 | 63.3 |
|  |  | 3 | 3.1 | 3.9 | 96.3 | 75.5 |
|  |  | 4 | 2.5 | 2.4 | 105 | 78.0 |
| dhSphingosine-1-P | 0.9961 | 1 | 7.1 | 17 | 107 | 75.8 |
|  |  | 2 | 8.3 | 8.5 | 94.9 | 72.3 |
|  |  | 3 | 7.3 | 9.7 | 85.8 | 73.9 |
|  |  | 4 | 2.6 | 5.5 | 102 | 66.6 |
| dhCer(d18:0/6:0) | 0.9986 | 1 | 1.4 | 4.3 | 114 | 71.6 |
|  |  | 2 | 3.8 | 5.0 | 101 | 75.5 |
|  |  | 3 | 2.6 | 3.7 | 95.5 | 66.1 |
|  |  | 4 | 1.1 | 2.2 | 104 | 63.9 |
| HexSph | 0.9900 | 1 | 3.6 | 3.6 | 118 | 67.5 |
|  |  | 2 | 2.8 | 5.1 | 91.1 | 68.0 |
|  |  | 3 | 8.9 | 12 | 98.2 | 79.7 |
|  |  | 4 | 4.5 | 8.4 | 111 | 80.5 |
| dhCer(d18:0/16:0) | 0.9976 | 1 | 3.4 | 4.7 | 112 | 63.3 |
|  |  | 2 | 3.0 | 9.8 | 106 | 65.8 |
|  |  | 3 | 3.5 | 9.8 | 97.2 | 63.8 |
|  |  | 4 | 2.4 | 6.6 | 117 | 74.6 |
| dhCer(d18:0/18:1) | 0.9973 | 1 | 4.1 | 9.4 | 94.2 | 66.5 |
|  |  | 2 | 4.5 | 4.8 | 93.7 | 67.5 |
|  |  | 3 | 3.0 | 10 | 93.3 | 80.0 |
|  |  | 4 | 2.1 | 6.3 | 117 | 70.2 |
| dhCer(d18:0/18:0) | 0.9943 | 1 | 2.6 | 5.2 | 114 | 75.2 |
|  |  | 2 | 3.4 | 7.1 | 89.7 | 73.8 |
|  |  | 3 | 3.9 | 15 | 80.8 | 80.4 |
|  |  | 4 | 4.3 | 6.8 | 105 | 74.4 |
| HexCer(d18:1/8:0) | 0.9948 | 1 | 1.5 | 2.7 | 105 | 79.3 |
|  |  | 2 | 2.5 | 4.5 | 90.3 | 73.6 |
|  |  | 3 | 2.2 | 5.9 | 87.4 | 68.8 |
|  |  | 4 | 2.3 | 4.8 | 102 | 64.7 |
| HexCer(d18:1/12:0) | 0.9956 | 1 | 5.3 | 13 | 91.7 | 67.6 |
|  |  | 2 | 3.8 | 13 | 85.5 | 68.8 |
|  |  | 3 | 5.2 | 9.5 | 94.1 | 67.4 |
|  |  | 4 | 4.7 | 9.1 | 96.7 | 75.5 |
| dhCer(d18:0/24:1) | 0.9926 | 1 | 3.9 | 12 | 107 | 66.2 |
|  |  | 2 | 4.9 | 4.5 | 93.5 | 65.7 |
|  |  | 3 | 3.9 | 10 | 112 | 72.8 |
|  |  | 4 | 3.0 | 7.2 | 105 | 69.0 |
| dhCer(d18:0/24:0) | 0.9910 | 1 | 23 | 16 | 107 | 69.7 |
|  |  | 2 | 11 | 7.5 | 102 | 64.5 |
|  |  | 3 | 1.9 | 15 | 83.6 | 73.0 |
|  |  | 4 | 8.9 | 12 | 103 | 67.8 |
| HexCer(d18:1/16:0) | 0.9911 | 1 | 2.0 | 11 | 105 | 72.0 |
|  |  | 2 | 1.9 | 12 | 116 | 71.6 |
|  |  | 3 | 3.3 | 7.1 | 99.2 | 75.0 |
|  |  | 4 | 1.8 | 4.3 | 112 | 76.7 |
| HexCer(d18:1/18:1) | 0.9943 | 1 | 5.7 | 9.8 | 112 | 71.1 |
|  |  | 2 | 4.0 | 11 | 113 | 71.8 |
|  |  | 3 | 3.5 | 7.7 | 108 | 77.4 |
|  |  | 4 | 2.1 | 11 | 106 | 70.5 |
| HexCer(d18:1/18:0) | 0.9963 | 1 | 2.6 | 4.7 | 117 | 66.0 |
|  |  | 2 | 1.9 | 8.0 | 116 | 78.5 |
|  |  | 3 | 3.5 | 11 | 105 | 80.3 |
|  |  | 4 | 2.3 | 6.2 | 120 | 81.0 |
| HexCer(d18:1/24:1) | 0.9912 | 1 | 2.4 | 19 | 83.0 | 69.7 |
|  |  | 2 | 3.6 | 12 | 91.9 | 75.5 |
|  |  | 3 | 3.7 | 11 | 107 | 74.1 |
|  |  | 4 | 6.9 | 12 | 106 | 74.2 |
| Lyso-SM(d18:1) | 0.9918 | 1 | 1.8 | 6.3 | 119 | 79.8 |
|  |  | 2 | 2.7 | 7.6 | 92.0 | 65.2 |
|  |  | 3 | 16 | 15 | 110 | 68.0 |
|  |  | 4 | 1.6 | 4.3 | 103 | 74.5 |
| SM(d18:1/2:0) | 0.9974 | 1 | 6.2 | 5.3 | 109 | 65.9 |
|  |  | 2 | 3.7 | 10 | 97.0 | 76.4 |
|  |  | 3 | 8.5 | 7.7 | 102 | 79.6 |
|  |  | 4 | 3.3 | 6.2 | 106 | 76.2 |
| SM(d18:1/6:0) | 0.9953 | 1 | 2.7 | 5.9 | 112 | 65.1 |
|  |  | 2 | 8.3 | 15 | 96.7 | 77.3 |
|  |  | 3 | 8.0 | 7.8 | 108 | 67.5 |
|  |  | 4 | 4.1 | 5.2 | 107 | 67.8 |
| SM(d18:1/12:0) | 0.9952 | 1 | 0.34 | 4.3 | 117 | 75.4 |
|  |  | 2 | 5.0 | 13 | 94.4 | 65.3 |
|  |  | 3 | 2.5 | 3.3 | 103 | 79.3 |
|  |  | 4 | 0.81 | 3.2 | 107 | 78.2 |
| SM(d18:1/16:0) | 0.9990 | 1 | 1.4 | 5.5 | 82.6 | 68.9 |
|  |  | 2 | 4.7 | 10 | 106 | 76.7 |
|  |  | 3 | 3.3 | 4.9 | 101 | 68.1 |
|  |  | 4 | 1.5 | 5.8 | 97.2 | 67.3 |
| SM(d18:1/18:1) | 0.9997 | 1 | 2.6 | 5.8 | 98.4 | 67.1 |
|  |  | 2 | 6.6 | 12 | 104 | 72.3 |
|  |  | 3 | 1.5 | 4.1 | 103 | 77.3 |
|  |  | 4 | 1.1 | 5.3 | 98.9 | 73.8 |
| SM(d18:1/18:0) | 0.9997 | 1 | 15 | 6.1 | 93.4 | 68.5 |
|  |  | 2 | 8.9 | 12 | 109 | 67.9 |
|  |  | 3 | 5.7 | 6.0 | 103 | 69.9 |
|  |  | 4 | 1.3 | 5.2 | 102 | 66.1 |
| SM(d18:1/24:1) | 0.9968 | 1 | 5.1 | 6.4 | 114 | 78.7 |
|  |  | 2 | 7.4 | 13 | 108 | 68.3 |
|  |  | 3 | 3.9 | 3.6 | 115 | 67.7 |
|  |  | 4 | 2.1 | 4.5 | 115 | 79.7 |
| SM(d18:1/24:0) | 0.9944 | 1 | 1.1 | 5.8 | 118 | 78.0 |
|  |  | 2 | 2.7 | 11 | 114 | 67.8 |
|  |  | 3 | 6.4 | 6.3 | 114 | 75.7 |
|  |  | 4 | 3.8 | 5.7 | 117 | 73.7 |

Data shown: linear correlation coefficient (r), intra-assay precision, inter-assays precision, accuracy, absolute recovery. Levels 1–4 represent 2.5, 10, 50, and 200 pmol/mg protein, respectively.

Abbreviations: IA Pre, Intra-assay precision; IE Pre, Inter-assay precision; A-Reco, absolute recovery.
