# Supplementary material for: Sphingolipids as New Biomarkers for Assessment of Delayed-Type Hypersensitivity and Response to Triptolide
Source: PLoS One. 2012 Dec 26;7(12):e52454. doi: 10.1371/journal.pone.0052454 (PMC3530451; doi:10.1371/journal.pone.0052454)
Supplement: Table S2 — Validation results for stock solution stability. (DOCX) [file pone.0052454.s005.docx]

**Table S2**. Validation results for stock solution stability

| Compound | 6 hours at room temperature | 60 days at -20ºC |
| --- | --- | --- |
|  | [%] | [%] |
| Sphingosine(d18:1) | 103 | 98.8 |
| Sphingosine(d17:1) | 100 | 100 |
| Cer(d18:1/2:0) | 104 | 99.2 |
| Cer(d18:1/4:0) | 99.2 | 95.4 |
| Cer(d18:1/6:0) | 103 | 101 |
| Cer(d18:1/8:0) | 101 | 98.7 |
| Cer(d18:1/10:0) | 95.2 | 98.6 |
| Cer(d18:1/12:0) | 101 | 99.3 |
| Cer(d18:1/14:0) | 99.0 | 99.2 |
| Cer(d18:1/16:0) | 98.0 | 99.3 |
| Cer(d17:1/18:0) | 100 | 99.0 |
| Cer(d18:1/18:1) | 101 | 101 |
| Cer(d18:1/18:0) | 101 | 97.2 |
| Cer(d18:1/20:0) | 104 | 98.2 |
| Cer(d17:1/24:1) | 104 | 99.7 |
| Cer(d18:1/22:0) | 104 | 97.8 |
| Cer(d18:1/24:1) | 106 | 97.1 |
| Cer(d18:1/24:0) | 108 | 98.0 |
| Sphingosine-1-P(d18:1) | 101 | 100 |
| Sphingosine-1-P(d17:1) | 96.1 | 102 |
| Cer(d18:1/2:0)-1-P | 97.7 | 96.0 |
| Cer(d18:1/8:0)-1-P | 101 | 100 |
| Cer(d18:1/12:0)-1-P | 95.7 | 98.2 |
| Cer(d18:1/16:0)-1-P | 95.5 | 99.5 |
| Cer(d18:1/18:1)-1-P | 97.4 | 100 |
| Lyso-SM(d18:1) | 95.1 | 99.6 |
| Lyso-SM(d17:1) | 96.1 | 94.4 |
| SM(d18:1/2:0) | 93.7 | 100 |
| SM(d18:1/6:0) | 94.0 | 99.6 |
| SM(d18:1/12:0) | 93.1 | 98.1 |
| SM(d18:1/16:0) | 103 | 101 |
| SM(d18:1/17:0) | 106 | 94.3 |
| SM(d18:1/18:1) | 104 | 99.3 |
| SM(d18:1/18:0) | 95.0 | 98.6 |
| SM(d18:1/24:1) | 93.9 | 96.2 |
| SM(d18:1/24:0) | 102 | 98.2 |
| HexSph(d18:1) | 97.2 | 94.6 |
| HexCer(d18:1/8:0) | 97.9 | 93.4 |
| HexCer(d18:1/12:0) | 96.2 | 92.4 |
| HexCer(d18:1/16:0) | 105 | 98.4 |
| HexCer(d18:1/18:1) | 92.2 | 98.7 |
| HexCer(d18:1/18:0) | 104 | 102 |
| HexCer(d18:1/24:1) | 92.7 | 105 |
| dhSph(d18:0) | 96.1 | 94.2 |
| dhSph(d17:0) | 102 | 96.0 |
| dhSph-1-P(d18:0) | 106 | 99.3 |
| dhSph-1-P(d17:0) | 106 | 104 |
| dhCer(d18:0/2:0) | 101 | 101 |
| dhCer(d18:0/6:0) | 99.9 | 100 |
| dhCer(d18:0/8:0) | 108 | 107 |
| dhCer(d18:0/16:0) | 93.9 | 99.3 |
| dhCer(d18:0/18:1) | 96.0 | 101 |
| dhCer(d18:0/18:0) | 93.4 | 103 |
| dhCer(d18:0/24:1) | 99.5 | 102 |
| dhCer(d18:0/24:0) | 97.8 | 97.8 |

To evaluate 6-hour stability, a freshly-made solution was analyzed after being kept at room temperature for 6 hours. To evaluate the 60-day stability, a stock solution was analyzed after being kept at -20ºC for 60 days. Stability was tested by comparing the peak area of the test sample with that of a freshly-prepared solution from the same batch.
