# Supplementary material for: Sphingolipids as New Biomarkers for Assessment of Delayed-Type Hypersensitivity and Response to Triptolide
Source: PLoS One. 2012 Dec 26;7(12):e52454. doi: 10.1371/journal.pone.0052454 (PMC3530451; doi:10.1371/journal.pone.0052454)
Supplement: Information S1 — Sample preparation: Procedures of sample preparation before HPLC-MS/MS. Standard curves and quality control samples: Preparation procedures of standard curve of each target compound and the quality control samples. Gradient elution programs: Gradient elution programs for each subclasses of sphingolipids. Method development: Key points about the development of this quantitative sphingolipidomic platform. They answered those following questions: 1) How were the chromatography and mass spectrometry conditions optimized? 2) Why we employed the segmental multiple reaction monitoring method on quantification of sphingolipids and what was the advantages? 3) How were the extraction method optimized? 4) What the usage of the unnatural sphingolipids detected by this platform? Method Validation: Procedures and results of the HPLC-MS/MS method validation. (DOCX) [file pone.0052454.s006.docx]

# SUPPORTING INFORMATION TEXT

## Sample preparation

0.1ml tissue homogenate correspond to 1mg protein or 0.1mL plasma was placed into a glass tube to which was added with 50μl internal standard (ISTD) in advance. 1.5 ml methanol was added to the tube, which was then vortexed. 5ml MTBE was then added and the tube was vortexed for 15 min. Phase separation was induced by adding 1.25 ml MS-grade water. After 10 min of incubation at room temperature, the tube was centrifuged at 1000 × g for 10 min. The organic supernatant was collected and the lower phase re-extracted with 2 ml of the solvent mixture (MTBE/methanol/water (10:3:2.5)) as above before acidifying with 0.1 ml formic acid. The supernatant was collected and the pooled organic supernatant was split into two parts, 2ml of which was dried under gentle nitrogen for sphingomyelin testing preparation while the rest of the supernatant was dried under gentle nitrogen. The residues of the rest supernatant were dissolved in 150 μl of mobile phase B before analysis.

The dried extracts of the 2ml extract were redissolved in 1 ml methanol and 50 µL of 2N NaOH in methanol and vortexed. The samples were allowed to undergo mild alkaline hydrolysis at 30ºC water bath for 3 h. After hydrolysis, the following solvents were added sequentially to complete the extraction: chloroform (1 ml), methanol (1 ml), 1N NaCl (0.8 ml), chloroform (1 ml), and 1N NaCl (1 ml). Samples were vortexed after the addition of each solvent and then centrifuged for 5 min at 3,000 g. The upper (aqueous) phase was aspirated including any inter-phase observed, then the lower (organic) phase was evaporated to dryness under gentle nitrogen. The dried residues were reconstituted in mobile phase B (150 µL) before analysis. Samples in HPLC auto-sampler vials were temporarily placed at 4°C until ready for HPLC-MS/MS system.

## Standard curves and quality control (QC) samples

Two sphingomyelin internal standards and other 12 sphingolipid internal standards as indicated in table 1 were prepared at a concentration of 4000 and 1000 pmol/ml respectively in methanol. The calibration mixtures except sphingomyelins for the standard curves were prepared at concentrations of 250 pmol/ml (Std A) and 2000 pmol/ml (Std B) in methanol. Sphingomyelin calibration mixtures for the standard curves were prepared at concentrations of 2000 pmol/ml (StdSM A) and 8000 pmol/ml (StdSM B).

The calibration curve standards were prepared using blank matrix, which was about 1 mg BSA dissolved in 0.1ml tissue homogenate buffer solution. For sphingosine, sphingosine-1-phosphate, ceramides, ceramides-1-phosphate, dihydrosphingosine, dihydorsphingosine-1-phosphate, dihydroceramides and hexosylceramides, all calibration curves comprised eight calibration points covering concentrations of 2.5, 5.0, 10.0, 20.0, 50.0, 100.0, 200.0 and 400.0 pmol/mg protein, and were constructed by spiking Std A or Std B. Quality control (QC) samples with low, middle and high concentration at 10.0, 50.0, 200.0 pmol/mg protein were also prepared in the way same as the calibration curves . For sphingomyelines, all calibration curves comprised eight calibration points covering concentrations of 12.5, 25.0, 50.0, 100.0, 250.0, 500.0, 1000.0 and 2000.0 pmol/mg protein, and were constructed by spiking StdSM A or StdSM B. QC samples with low, middle and high concentration at 50.0, 250.0, 1000.0 pmol/mg protein were also prepared in the way same as the calibration curves. All calibration curves were constructed by the peak area ratios of the analyte to its corresponding internal standard against the eight calibration point concentration[[1](#_ENREF_1)]. For detailed information about method development and method validation, please refer to supplementary information, item 1.4 and 1.5, respectively.

## Gradient elution programs

For sphingosine, sphingosine-1-phosphate, ceramides and ceramides-1-phosphate, the gradient elution was consecutively programmed as follows: t=0 – 10 min: A (15%) and B (85%); t=10 – 11 min: A (15–8%) and B (85–92%); t=11 – 25 min: A (8–3%) and B (92–97%); t=25 – 42 min: A (3–1%) and B (97–99%). The detection time for segmental multiple reaction monitoring was divided into 4 time windows by cutting off at the time of 11.0 min, 23.0min and 28.0 min.

For dihydrosphingosine, dihydrosphingosine-1-phosphate, dihydroceramides and hexosyl-ceramides, the gradient elution was consecutively programmed as follows: t=0 – 7 min: A (18%) and B (82%); t=7 – 12 min: A (18–8%) and B (82–92%); t=12 – 18 min: A (8–4%) and B (92–96%); t=18 – 25 min:A (4–1%) and B (96–99%); t=25 – 35 min:A (1%) and B (99%). The detection time for segmental multiple reaction monitoring was divided into 3 time windows by cutting off at the time of 18.2 min and 25.9min.

For sphingomyelins, the gradient elution was consecutively programmed as follows: t=0 – 5 min A (10–1%) and B (90–99%); t=5 – 15 min A (1%) and B (99%). The detection time for segmental multiple reaction monitoring was divided into 2 time windows by cutting off at the time of 6.5 min.

The post time was set at 6 min to allow column equilibrium. The injection volume was 10 μl and the column temperature was maintained at 30°C.

## Method development

### Optimization of chromatography and mass spectrometry conditions

Other than analyzing common lipids with high abundance in mammalian using non-targeted shotgun methods[[2](#_ENREF_2)], analyzing sphingolipids needs a more sensitive method, such as MRM method which is by far one of the most sensitive method for quantification. LC-MS/MS is powerful technique for sphingolipidomics. The MRM scan mode commonly makes it unnecessary to separate all the sphingolipids. However, this is not always the truth. For example, in the present study, Cer(d18:1/18:0) and Cer(d18:1/18:1) showed the similar MS/MS fragmentation pattern and their precursor ions were very close (566.4 and 564.4), as were the product ions (546.2 and 548.3). Therefore, the ^13^C isotopes in the Cer(d18:1/18:1) lipid yielding the smaller molecular ion interfered significantly with the quantification of Cer(d18:1/18:0) if they were not separated on column. Such inference was also observed for Cer(d18:1/24:0) and Cer(d18:1/24:1) (Figure S3). Whether those compounds would separate on the column dictated whether this quantification method would be successful. In addition, due to the limitation of mass scan rate and resolution, loss of sensitivity that is essential for quantification of some low-abundance sphingolipids is unavoidable if the target sphingolipids are not well resolved on column and scanned in a single LC-MS/MS run. On the other hand, the method of sample preparation and the concentrations of different sphingolipids are very different in biological samples, such as sphingomyelins, abundance of which are hundreds times as other sphingolipids. During the extraction of sphingomyelin, elimination of phosphatidylcholine by alkaline hydrolysis was necessary to avoid the inference caused by the exactly same retention time and MRM characteristics between sphingomyelin and phosphatidylcholine[[3](#_ENREF_3)]. Due to the relatively slow scan rate of our mass spectrometer and also to gain better accuracy and sensitivity we divided 43 target compounds into 3 different LC-MS/MS runs according to their subclasses (Table 1).

To develop a method with the desired LLOQ (166 fmol on-column) and selectivity, it was necessary to use MS/MS detection methods. Precursor ion, product ion, fragmentor and collision energies are the four key parameters to be optimized to gain stable and the best response of each compound on triple quadpole mass spectrometer. First, the fragmentor energy was optimized to ensure that the [M+H]^+^ was the predominant ion in the Q1 spectrum, and also to obtain the strongest signal with a range between 60 and 240V. Second, the collision energy was optimized to ensure that the inferential product ion produced the best signal; however, according to Jian Wang et al.[[4](#_ENREF_4)], the highest response may not always give the best result. The parameter with the highest signal may not give the most stable response when injecting the same sample consecutively. Therefore, we tested each parameter six times in succession, and calculated the relative standard deviation (RSD) of signal response for each compound. The parameter showing the best response and an RSD < 10% was chosen. In general m/z 264 and m/z 266 were the product ions for ceramides and dihydroceramides respectively by the loss of the N-acyl chain in the positive ion mode [[5](#_ENREF_5),[6](#_ENREF_6)]. They both are essential for the identifications of ceramides and dihydroceramides. However in this study, the single dehydration for ceramide's and dihydroceramide’s MRM transition provided the stable and best signal response and was chosen for quantification. Moreover above ceramide's and dihydroceramide’s MRM transition has been used in other studies [[3](#_ENREF_3)]. As long as the parent ions and product ions of MRM transitions offered better snesitivity , specificity and stable signal response of each compound, they were suitable for the quantification. It should be reliable enough to use the confirmed MRM transition and the retention time same as the structure-confirmed standards to qualitative identify the target compounds.

### Segmental multiple reaction monitoring on quantification of sphingolipids

In the present study, the mass analysis protocol was divided into separate segments to improve the sensitivity because of the limited scan rate of the mass spectrometer (Table 1). The segments of mass detection were determined according to the gap between two peaks and the gradient of the mobile phase. There could be a segment once the gap was more than 0.5 minutes. According to Liang's research[[7](#_ENREF_7)], the less MRM transitions in a single segment the better sensitivity we can get because of the more scan times, better baseline and less inferences on the chromatograph. In addition, such operation could dramatically broaden their dynamic ranges. By employing segmental MRM method for sphingolipids, we found it was sensitive enough for mapping the main metabolic pathway of sphingolipids.

### Optimization of the extraction method

Recently, Matyash et al. reported a new lipid extraction method based on a methyl-tert-butyl ether (MTBE)/methanol/water solvent system specifically developed for lipidomics research from excessive kinds of biological samples[[8](#_ENREF_8)]. Lipids are recovered into the MTBE phase, which, because of its lower density, comprises the upper phase of the two-phase solvent system. As a result, contamination (which can be a problem with the Folch method and the Bligh & Dyer method) is avoided[[9](#_ENREF_9),[10](#_ENREF_10)]. Thus, based on the work of Matyash and our own optimization studies, the combination of MTBE and methanol containing 5% formic acid and water (20:6:5 v/v/v) was chosen as some target sphingolipids such as ceramide-1-phosphate and sphingosine-1-phosphate were acid compounds. The recovery of all target sphingolipids from the spiked matrix samples was consistent, precise and reproducible.

A good internal standard should mimic the analytes over the entire sample preparation procedure, even in the responses observed in mass spectrometry. It should track the analyte during the extraction and compensate for any potential inconsistencies in recovery. Stable isotopes of a target compound are ideal candidates for meeting the above criteria; however, isotopes are not always easily accessible because of their high cost and technical difficulties experienced when synthesizing them. Therefore, for biological sample analysis, non-natural chemical compounds with a similar structure to that of the target compound is always the first choice. We did not find proper ISTD with C17 sphingosine base for monohexosylceramide from Avanti or Sigma. In some research they use C8-GlcCer or C12-GlcCer as the ISTD[[3](#_ENREF_3)]. However, in our studies we find that such ISTD compounds could be detected in tissues of mice above the LOD and below the LOQ. We considered such compounds might not be proper ISTDs for quantification of monohexosylceramide. Also according to the calibration and QC test, ceramide ISTDs were suitable for the quantification of monohexosylceramide because of the similar extraction recovery and MS response. It should be emphasized that, during the extraction, all the containers (including the tubes used to prepare the working solutions and samples) must be made from glass. Because of the weak polarity of the extraction solvents, some substances in the plastic used to make the tubes can be extracted into the solvents, and might result in matrix contamination and leaks of foreign compounds into the sample.

### Unnatural sphingolipids

This study reports the development of three quantification methods for 43 sphingolipids, some of which do not occur naturally. Because both natural and non-natural sphingolipids have similar structures, one can avoid interference from natural sphingolipids by introducing non-natural sphingolipids into cell lines. Due to the very high hydrophobicity and poor solubility of natural long-chain ceramides sphingolipids, It is difficult to deliver them to cells. One of the major approaches to overcome this problem is the use of short-chain ceramides, which can be quite easily dissolved in ordinary solvents[[11](#_ENREF_11)]. The function and metabolism of these non-natural sphingolipids has previously been studied by monitoring their effects after adding them to cell lines[[12](#_ENREF_12),[13](#_ENREF_13)]. Thus, we decided to include these common non-natural sphingolipids such as Cer(d18:1/4:0), Cer(d18:1/6:0), Cer(d18:1/8:0), Cer(d18:1/2:0)-1-P, dhCer(d18:0/2:0), dhCer(d18:0/6:0), HexCer(d18:1/8:0), SM(d18:1/2:0), SM(d18:1/6:0) in our study.

## Method validation

A HPLC-MS/MS method for analyzing sphingolipids in biological samples was developed and validated according to the principles of Good Laboratory Practice and the Guidance of Industry Bioanalytical Method Validation [[1](#_ENREF_1),[14](#_ENREF_14)]. The validation consisted of specificity, linearity, absolute recovery, precision, accuracy and stability. The method was validated over a concentration range of 2.5–400 pmol/mg protein for sphingomyelins and 12.5–2000pmol/mg protein for other sphingolipids, respectively. Samples were prepared using a two-phase extraction with MTBE, methanol, and water. The reconstituted samples were analyzed by LC-MS/MS using a Spectra C8SR column and an Agilent 6410B Triple Quad mass spectrometer. The retention times for the different sphingolipids are listed in Table 1. The MS/MS fragmentation patterns of representative compounds are shown in Figure S1. No significant direct interference by other compounds was observed in biological samples including spleen, kidney, liver and blood. Representative MRM chromatograms including the LLOQ sample of each method and the sample of mice spleen (~1mg protein) are shown in Figure S2**.** Linearity evaluated using a weighted least-squares regression analysis of an eight point standard curve. The calibration curves were generated from peak area ratios of the analyte to IS by weighted (1/x) least-squares linear regression. Examination of extended calibration ranges indicates (data not shown) that linear MS response can be extended up to 5000 for sphingomyelins and 2000 pmol for other sphingolipids, respectively. The linear correlation coefficient (r) was consistently greater than 0.99 during the validation. The absolute recovery was greater than 60% and consistent for all target compounds and internal standards. Precision and accuracy tests showed that the method could deliver accurate and precise results for ordinary biological analysis within the linear range mentioned above. The stability tests proved that the treated and untreated biological samples, the standard solutions were stable enough during the certain period time, which satisfied routine analysis. Validation result is shown in Table S1 and Table S2.

# REFERENCES

1. Committee BC (2001) Guidance for Industry Bioanalytical Method Validation. In: Services USDoHaH, Administration FaD, Research CfDEa, Medicine CfV, editors.

2. Han X, Yang K, Gross RW (2012) Multi-dimensional mass spectrometry-based shotgun lipidomics and novel strategies for lipidomic analyses. Mass Spectrom Rev 31: 134-178.

3. Bielawski J, Pierce JS, Snider J, Rembiesa B, Szulc ZM, et al. (2009) Comprehensive quantitative analysis of bioactive sphingolipids by high-performance liquid chromatography-tandem mass spectrometry. Methods Mol Biol 579: 443-467.

4. Wang J, Aubry A, Bolgar MS, Gu H, Olah TV, et al. (2010) Effect of mobile phase pH, aqueous-organic ratio, and buffer concentration on electrospray ionization tandem mass spectrometric fragmentation patterns: implications in liquid chromatography/tandem mass spectrometric bioanalysis. Rapid Commun Mass Spectrom 24: 3221-3229.

5. Merrill AH, Jr., Sullards MC, Allegood JC, Kelly S, Wang E (2005) Sphingolipidomics: high-throughput, structure-specific, and quantitative analysis of sphingolipids by liquid chromatography tandem mass spectrometry. Methods 36: 207-224.

6. Yoo HH, Son J, Kim DH (2006) Liquid chromatography-tandem mass spectrometric determination of ceramides and related lipid species in cellular extracts. J Chromatogr B Analyt Technol Biomed Life Sci 843: 327-333.

7. Liang Y, Kang A, Xie T, Zheng X, Dai C, et al. (2010) Influence of segmental and selected ion monitoring on quantitation of multi-component using high-pressure liquid chromatography-quadrupole mass spectrometry: Simultaneous detection of 16 saponins in rat plasma as a case. J Chromatogr A 1217: 4501-4506.

8. Matyash V, Liebisch G, Kurzchalia TV, Shevchenko A, Schwudke D (2008) Lipid extraction by methyl-tert-butyl ether for high-throughput lipidomics. J Lipid Res 49: 1137-1146.

9. Folch J, Lees M, Sloane Stanley GH (1957) A simple method for the isolation and purification of total lipides from animal tissues. J Biol Chem 226: 497-509.

10. Bligh EG, Dyer WJ (1959) A rapid method of total lipid extraction and purification. Can J Biochem Physiol 37: 911-917.

11. Luberto C, Hannun YA (2000) Use of short-chain ceramides. In: Alfred H. Merrill JYAH, editor. Methods in Enzymology: Academic Press. pp. 407-420.

12. Zhu QY, Wang Z, Ji C, Cheng L, Yang YL, et al. (2011) C6-ceramide synergistically potentiates the anti-tumor effects of histone deacetylase inhibitors via AKT dephosphorylation and alpha-tubulin hyperacetylation both in vitro and in vivo. Cell Death Dis 2: e117.

13. Yu T, Li J, Sun H (2010) C6 ceramide potentiates curcumin-induced cell death and apoptosis in melanoma cell lines in vitro. Cancer Chemother Pharmacol 66: 999-1003.

14. Organization TWH (2009) Handbook: Good laboratory practice (2nd edition).
